# Silicon Modulates the Expression of Copper-Responsive Transporters and Non-Coding RNAs in Cucumber Subjected to Copper Stress

**DOI:** 10.3390/plants15203189

**Published:** 2026-10-18

**Authors:** Dragana Bosnić, Gordana Timotijević, Dragana Nikolić, Jelena Samardžić

**Affiliations:** Institute of Molecular Genetics and Genetic Engineering, Department of Microbiology and Plant Biology, University of Belgrade, Vojvode Stepe 444a, 11042 Belgrade, Serbia; dragana.bosnic@imgge.bg.ac.rs (D.B.); gordana.timotijevic@imgge.bg.ac.rs (G.T.); dragana.nikolic@imgge.bg.ac.rs (D.N.)

**Keywords:** silicon, copper stress, transporters, non-coding RNAs, miR398–SOD, cucumber

## Abstract

Excess copper (Cu) disrupts metal homeostasis and induces oxidative stress in plants. Silicon (Si) is known to mitigate heavy metal toxicity, yet the molecular basis of its protective role under Cu stress remains insufficiently understood. To address this, an exploratory transcriptome-wide screening was performed to identify candidate differentially expressed genes (DEGs), long non-coding RNAs (lncRNAs), and microRNAs responsive to excess Cu and/or Si in cucumber (*Cucumis sativus* cv. Chinese Long). Genes encoding metal transporters and selected miRNA–target pairs were further analyzed by qRT-PCR. Silicon alone modulated the expression of numerous candidate DEGs, notably enhancing the expression of metal transporter genes. Exposure to excess Cu suppressed genes involved in metal uptake while upregulating those associated with efflux and metal redistribution. Application of Si under Cu stress mitigated these transcriptional alterations. Cu stress repressed miR398 and miR408, concomitantly increasing the expression of their targets: superoxide dismutase (*SOD*) and plantacyanin (*PCY*). Furthermore, excess Cu downregulated miR390, resulting in increased expression of its predicted lncRNA target. The addition of Si to Cu-stressed plants further enhanced *SOD* expression while attenuating the upregulation of *PCY* and the lncRNA. The results demonstrated that Si modulates both coding and non-coding RNA expression under optimal and Cu-stress conditions, revealing post-transcriptional gene expression regulation as an additional layer of Si’s effect on plants. When integrated with previously reported physiological and biochemical data, these findings advance our understanding of the molecular basis of Si-mediated heavy metal tolerance in plants.

## 1. Introduction

Copper is an essential micronutrient for fundamental plant processes such as photosynthesis and respiration, serving as a cofactor in various Cu-containing proteins. However, excess Cu causes phytotoxicity at morphological, physiological, and molecular levels. The toxic effects primarily manifest in the root system due to the excessive accumulation of Cu in this organ [1]. Plants exhibit a range of physiological responses to Cu toxicity, underscoring the complexity of maintaining Cu ion homeostasis in plant cells. A network of Cu transporters plays a critical role in this regulation by facilitating processes such as Cu uptake, chelation, transport, and compartmentalization. These transporters are broadly classified into two groups: uptake transporters, which move Cu ions from the external environment into plant cells, and efflux transporters, which transport Cu ions either out of the cell or into subcellular compartments [2,3]. The COPT (COPper Transporter) family comprises highly conserved proteins found in all eukaryotes [4]. Among them, COPT1 is essential for root Cu uptake in dicots and exhibits high specificity for the reduced Cu^+^ form [5]. Cu uptake is preceded by the reduction of Cu^2+^ at the plasma membrane by metal reductases of the FRO (Ferric Reductase Oxidase) family. In addition to specific Cu transporters, plants also utilize non-specific divalent metal transporters, such as members of the ZIP (Zrt/Irt-like Protein) family including Iron-Regulated Transporter 1 (IRT1), and NRAMP (Natural Resistance-Associated Macrophage Proteins). These proteins have limited ion specificity and can transport a range of divalent metal ions across cell membranes, including Cu [6,7,8]. Vacuolar compartmentalization of Cu involves tonoplast transporters such as HMA5 (Heavy Metal ATPase 5) and COPT5, which transport Cu into and out of the vacuole, respectively. COPT5 is highly expressed in roots and facilitates Cu mobilization from root stores to photosynthetic and reproductive organs [9,10,11]. HMA5 also mediates Cu transport to the aboveground parts of plants by loading Cu into the xylem. Long-dist ance transport of Cu via the xylem and phloem involves oxidation of Cu^+^ to Cu^2+^ and complexation with metal-chelating agents, notably nicotianamine (NA), histidine, and organic acids [12]. The YSL (Yellow Stripe-Like) transporters are recognized as key regulators of the internal transport and distribution of metal ions in plants. YSL3 mediates the transport of metal–NA complexes and supports long-distance metal redistribution. Specifically, *AtYSL2* and *AtYSL3* from *Arabidopsis thaliana* facilitate lateral Cu movement within the vascular bundles of roots and stems [13].

The expression of genes involved in Cu homeostasis is tightly regulated at both transcriptional and post-transcriptional levels. Transcriptional regulators include SQUAMOSA Promoter binding protein-Like7 (SPL7) [14], whereas post-transcriptional regulation involves non-coding RNAs (ncRNAs), such as microRNAs (miRNAs) and long non-coding RNAs (lncRNAs) [15]. miRNAs are small RNAs (~21–24 nucleotides) that regulate gene expression through translational repression or by cleaving target mRNAs via the RNA-induced silencing complex, with the Argonaute 1 (AGO1) protein as a core component. miRNAs have critical roles in various developmental processes and in responses to environmental stresses, including drought (e.g., miR156, miR169), salinity (e.g., miR393), heavy metal stress (e.g., miR168, miR390), and nutrient deficiency (e.g., miR399). Even short-term changes in miRNA expression during stress can lead to significant physiological effects [16]. Several miRNAs, termed Cu-responsive miRNAs, alter their expression in response to changes in cellular Cu concentration [14]. Among them, miR398 and miR408 are highly conserved across plant species. Their known targets include non-essential Cu-binding proteins, such as copper/zinc (Cu/Zn) superoxide dismutase (SOD) and members of the phytocyanin family, including plantacyanin and laccases [17,18,19]. Cu/Zn SOD is a part of the antioxidative defense system. Plantacyanin (PCY) is a small blue copper protein implicated in redox-related processes and intercellular signaling. Although its precise physiological function remains unresolved, its extracellular localization suggests a potential role in modulating redox conditions in the apoplast, a critical interface for metal exchange, reactive oxygen species production, and cell wall remodeling under stress conditions [20]. The downregulation of these targets under Cu-deficiency, mediated by miR398/miR408, enables the preferential allocation of available Cu to essential Cu-binding proteins in the plant when the supply of this element is limited [21]. However, their regulatory dynamics under Cu excess remain largely unknown. Some evidence suggests that Cu-responsive miRNAs may also fine-tune the targets’ expression as part of a broader mechanism to balance Cu distribution and limit oxidative damage [18].

Long non-coding RNAs (lncRNAs) are transcripts longer than 200 nucleotides that do not encode proteins and represent an additional layer of gene regulation. LncRNAs exhibit tissue-specific expression, low abundance, and limited conservation across different species. They regulate gene expression both positively and negatively and function in cis- and trans-regulatory contexts. Although plant lncRNAs are not yet fully understood, emerging data have revealed their involvement in root development, photomorphogenesis, and responses to various stresses [22,23]. Recent studies in cucumber have demonstrated that specific root-expressed lncRNAs are associated with hypoxia tolerance acquired through priming [24].

Silicon (Si) is recognized as a beneficial element for plants, capable of mitigating heavy metal stress and decreasing its accumulation in plant tissue [25]. In rice fed with Si, decreased accumulation of toxic cadmium (Cd), manganese (Mn) and zinc (Zn) was observed, along with downregulation of transporter genes involved in their uptake and translocation [26,27,28]. In cucumber, Si enhanced Cu tolerance by stimulating the production of amino acids such as nicotianamine and histidine, which effectively chelate excess Cu [29]. Additionally, increased accumulation of Cu-binding proteins, which can buffer excessive Cu ions, has been observed in Si-fed cucumber plants [30]. However, the effect of Si on gene expression related to Cu metabolism remains poorly understood. Limited data suggest that Si may influence this regulatory level, as demonstrated in *A. thaliana* [31,32]. To date, expression profiling of miRNAs and lncRNAs under combined Cu and Si treatment has not been reported.

The aim of this study was to investigate the effect of Si supplementation on the expression of genes involved in Cu uptake and transport in cucumber plants exposed to Cu stress. Special emphasis was placed on the expression of miR398 and miR408 and their targets (SOD and PCY), as well as an lncRNA with unknown function. The results highlight the detoxification mechanisms in cucumber roots, the primary site of Cu accumulation. Furthermore, the potential effects of Si on shaping the transcriptome and miRNAome of cucumber plants are discussed.

## 2. Results

### 2.1. Transcriptome Analysis

**Figure 1:**
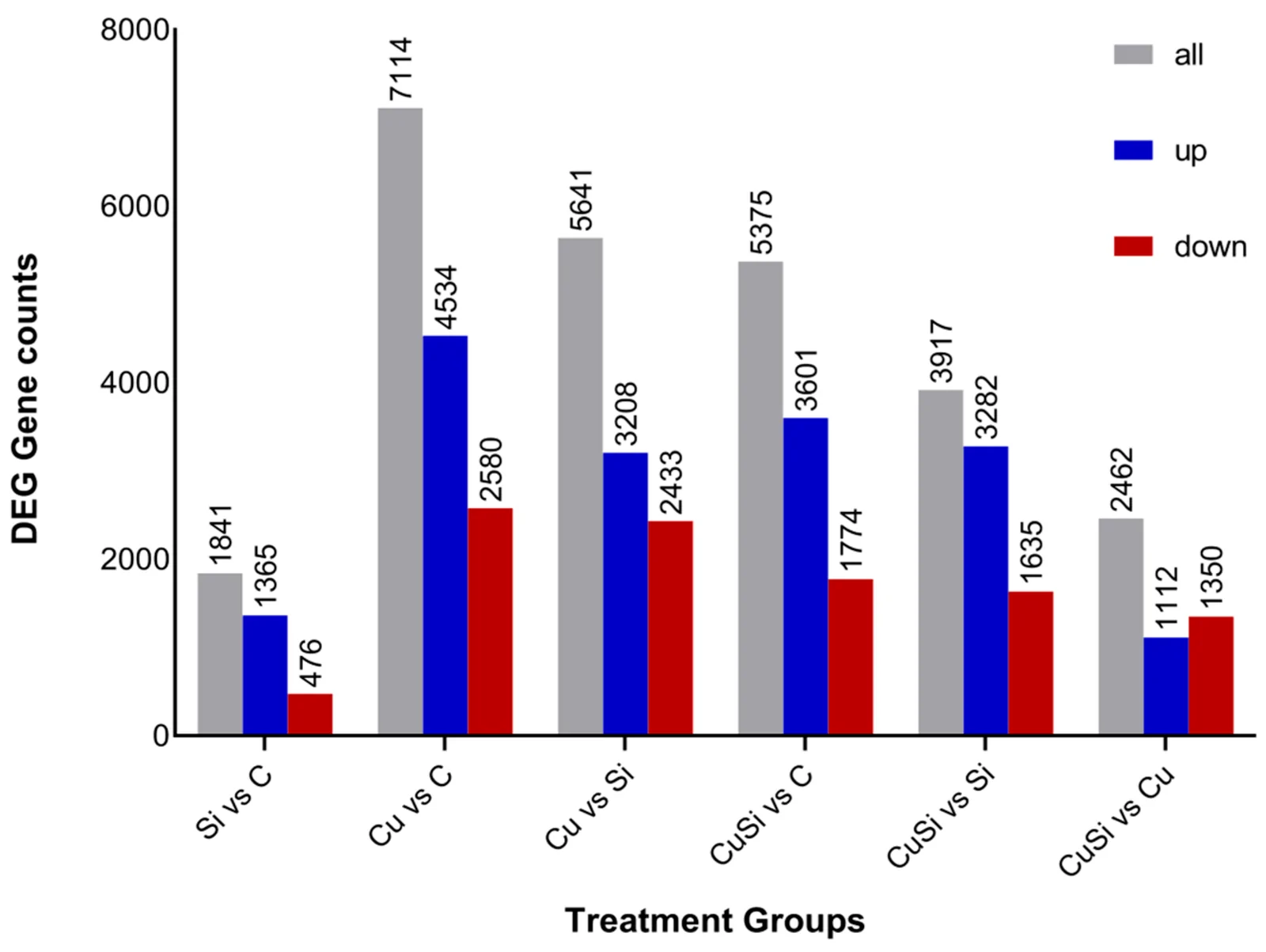
Distribution of candidate differentially expressed genes (DEGs) across pairwise treatment comparisons. Plants were grown under optimal Cu concentrations (C) or treated with 1.5 mM silicon (Si), 10 µM Cu (Cu), or co-treated with 10 µM Cu and 1.5 mM Si (CuSi). Samples were analyzed after five days of treatment.

To gain comprehensive insight into Cu- and Si-induced transcriptomic changes and their association with non-coding RNA expression, we performed an exploratory transcriptome-wide screening of mRNAs, miRNAs, and lncRNAs in cucumber roots after five days of Cu and/or Si treatment. The transcriptomic results were considered as screening data, and the identified candidate DEGs and enrichment analyses were used primarily to generate hypotheses and guide subsequent validation experiments. The transcriptomic response of cucumber roots was assessed by analyzing 23,998 transcripts, of which 14,743 were commonly expressed across all four treatments (Figure S1). Cluster analysis identified candidate differentially expressed genes (DEGs) and grouped those with similar expression profiles into four subclusters. Compared to Cu treatment, CuSi treatment tended to revert the exploratory transcriptome profile to a state more similar to that of control plants (Figure S2). Although the data were considered screening data, further analysis of candidate DEGs was conducted for each comparison group. The greatest number of candidate DEGs was observed under Cu treatment compared to the control (Figure 1).

### 2.2. Identification of Candidate Differentially Expressed (DE) lncRNAs

**Figure 2:**
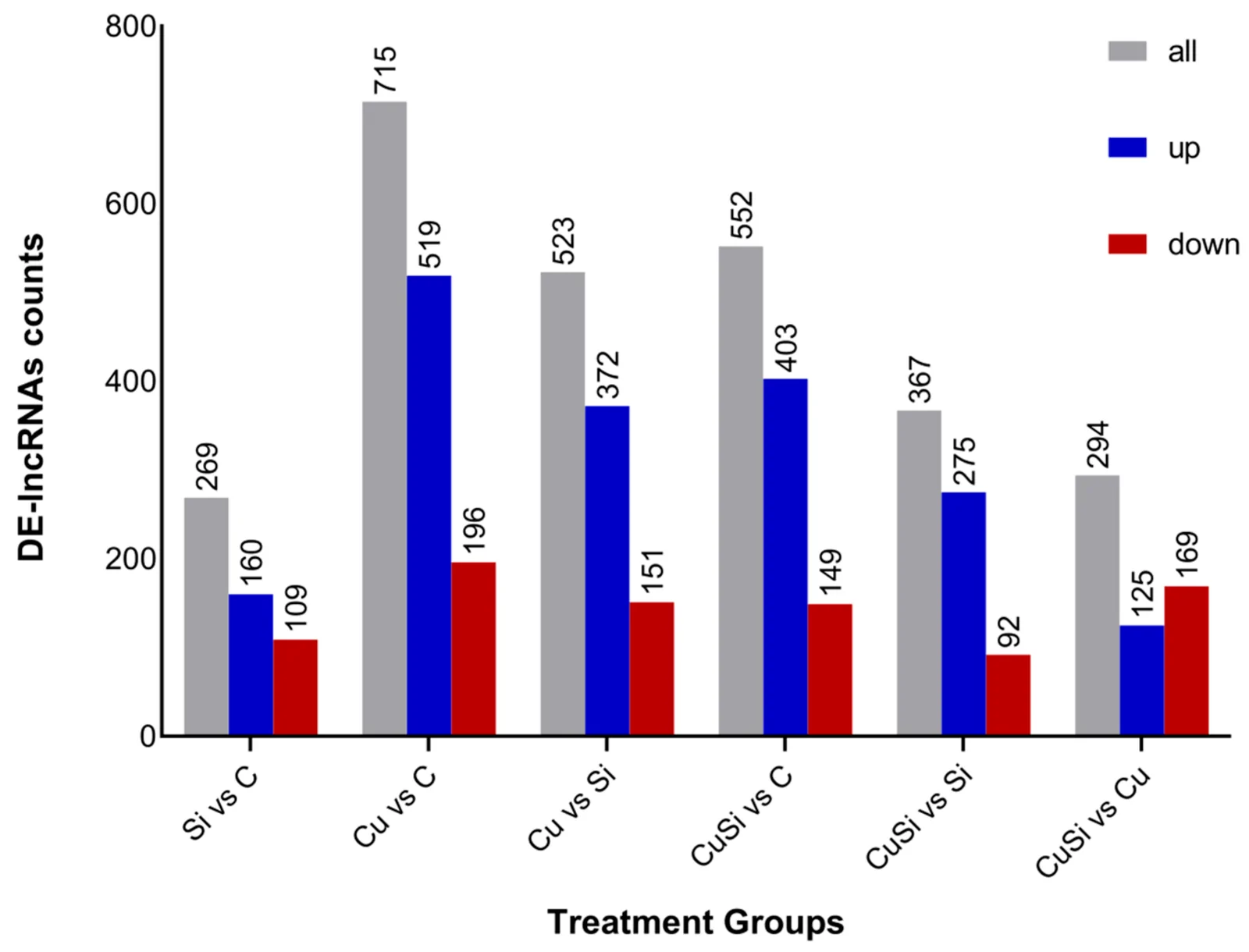
Distribution of candidate differentially expressed lncRNAs (DE-lncRNAs) across pairwise treatment comparisons. Plants were grown under optimal Cu concentrations (C) or treated with 1.5 mM silicon (Si), 10 µM Cu (Cu), or co-treated with 10 µM Cu and 1.5 mM Si (CuSi). Samples were analyzed after five days of treatment.

Among the 23,998 identified transcripts in the cucumber transcriptome, approximately 10% (2453) were long non-coding RNAs (lncRNAs). The majority of lncRNAs (>50%) ranged from 400 to 1000 bp in length, while the second-largest group comprised lncRNAs longer than 2000 bp (approximately 11%) (Figure S3). Exploratory differential expression analysis revealed that Cu treatment led to the highest number of candidate differentially expressed (DE) lncRNAs compared with the control (Figure 2).

### 2.3. Functional Classification of Candidate DEGs

As an exploratory approach, Gene Ontology (GO) classification and Kyoto Encyclopedia of Genes and Genomes (KEGG) pathway enrichment analyses were conducted to identify biological processes and pathways potentially associated with the silicon response. GO analysis revealed that candidate DEGs were predominantly associated with molecular functions and biological processes. Approximately 15 GO terms were significantly enriched across all treatments (Table 1). The most prevalent GO terms in the biological process category were “response to stimulus” and “carbohydrate metabolic process.” In the cellular component category, the “extracellular region” was the most frequently identified GO term. The most common molecular function GO term across all comparison groups was “DNA binding transcription factor activity.” The most abundant GO term in terms of candidate DEG enrichment was “transmembrane transporter activity.”

**Figure 3:**
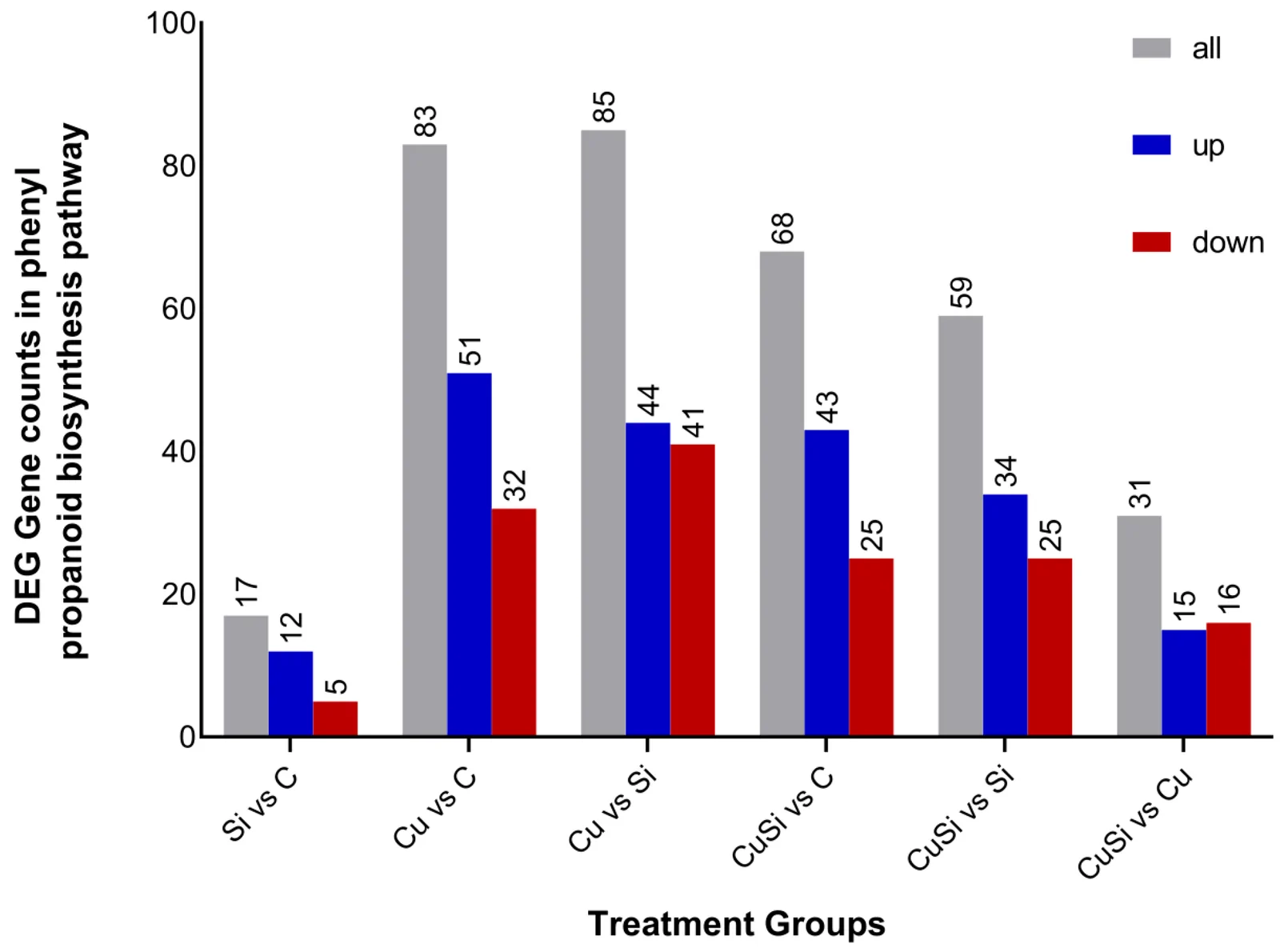
Distribution of candidate differentially expressed genes (DEGs) associated with the phenylpropanoid biosynthesis pathway identified by exploratory KEGG analysis. Plants were grown under optimal Cu concentrations (C) or treated with 1.5 mM silicon (Si), 10 µM Cu (Cu), or co-treated with 10 µM Cu and 1.5 mM Si (CuSi). Samples were analyzed after five days of treatment.

KEGG pathway enrichment analysis highlighted the phenylpropanoid biosynthesis pathway as the most significantly enriched (corrected *p*-value < 0.01) pathway, exhibiting more upregulated than downregulated genes in the Cu vs. control, CuSi vs. control, Cu vs. Si, and CuSi vs. Si comparisons. When comparing CuSi to Cu, the phenylpropanoid biosynthesis pathway remained the most significantly enriched, but more genes were downregulated than upregulated (Figure 3).

### 2.4. Temporal Dynamics of Transporter Gene Expression

**Figure 4:**
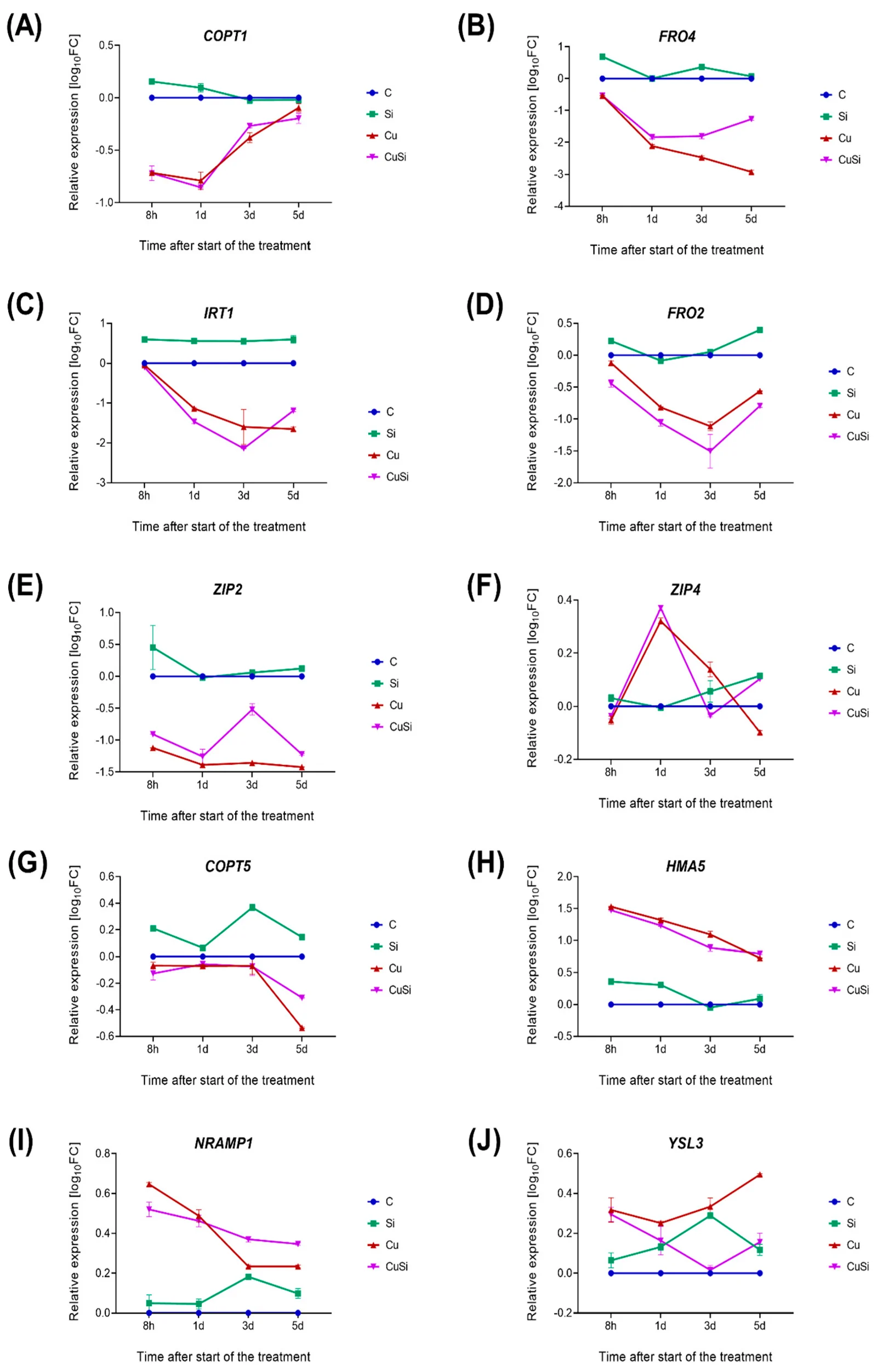
Relative gene expression of metal transporters in cucumber roots under Cu and/or Si treatments: *COPT1* (**A**), *FRO4* (**B**), *IRT1* (**C**), *FRO2* (**D**), *ZIP2* (**E**), *ZIP4* (**F**), *COPT5* (**G**), *HMA5* (**H**), *NRAMP1* (**I**), *YSL3* (**J**). Plants were grown under optimal Cu concentrations (C) or treated with 1.5 mM silicon (Si), 10 µM Cu (Cu), or co-treated with 10 µM Cu and 1.5 mM Si (CuSi) and analyzed after 8 h, one (1 d), three (3 d), or five (5 d) days after the beginning of the treatment. The ordinate shows gene expression as log_10_ FC (fold change). Data are presented as the mean log_10_FC of values obtained in repeated experiments (*n* = 3). Error bars indicate propagated SD (standard deviation). For statistical analysis (Table S3), two-way ANOVA with treatment as the primary factor followed by Tukey’s multiple comparison test was performed using GraphPad Prism 8.0. log_10_FC and propagated SD values for all treatments are provided in Supplementary Table S4.

To gain insight into the dynamics of Cu and Si effects on transporter gene expression, we conducted a time-course analysis of key genes involved in metal uptake, including *COPT1*, *IRT1*, and *NRAMP1*; the reductases *FRO4* and *FRO2*; and genes involved in internal metal transport, including *YSL3*, *COPT5*, and *HMA5*. After 8 h of Si treatment, *COPT1* levels were elevated and then gradually declined, returning to control levels by day 5 (Figure 4A). In plants exposed to Cu, *COPT1* expression began at very low levels but steadily increased over time. The CuSi treatment produced a temporal profile similar to that of Cu, though by day 5 the expression level was lower than in the Cu treatment. Similarly, Si stimulated *FRO4* expression (Figure 4B). In contrast, Cu exposure completely suppressed *FRO4*, keeping the gene downregulated throughout the experiment. The CuSi treatment initially resembled the Cu treatment profile; however, from day 1 onward, *FRO4* expression rose and continued to increase until day 5. Silicon treatment also strongly upregulated *IRT1* expression, with transcript levels markedly exceeding those of all other treatments and the control (Figure 4C). In contrast, Cu rapidly suppressed *IRT1*, and expression continued to decline from day 1 onward. Notably, the stimulatory effect of Si was completely abolished in the presence of Cu, as the CuSi treatment profile closely mirrored that of Cu, although reaching higher values on day 5 than the Cu treatment. Silicon exhibited a moderate stimulatory effect on *FRO2* expression (Figure 4D). In contrast, Cu acted as a potent inhibitor, decreasing *FRO2* expression and maintaining its suppression throughout the study. Notably, the CuSi combination resulted in even stronger suppression of the gene. Furthermore, Si induced the upregulation of *ZIP2* expression within 8 h (Figure 4E). Following this initial surge, expression levels returned close to the control by day 1. In contrast, Cu treatment caused prominent suppression of *ZIP2* throughout the experiment. Similarly, the combined CuSi treatment led to suppression of *ZIP2*, though less pronounced compared to Cu treatment. Regarding *ZIP4* expression, Si treatment maintained a stable profile mirroring the control, with no significant fluctuations throughout the period, except for an elevation on day 5 (Figure 4F). In contrast, Cu led to a sharp induction of expression one day after treatment onset, followed by a marked decline, with expression reaching levels below the control on day 5. A similar temporal pattern was observed in the CuSi treatment; however, the expression peak on day 1 was even more prominent than in the Cu treatment and remained elevated on day 5.

Furthermore, Si increased *COPT5* expression (Figure 4G). In contrast, Cu consistently suppressed the gene, resulting in a marked decline by day 5. The CuSi treatment profile largely followed the Cu treatment trend; however, expression increased relative to the Cu treatment at the final time point. All treatments upregulated *HMA5* (Figure 4H). Silicon treatment showed a slight increase compared with the other two treatments. Cu induced robust upregulation of *HMA5*, which, although sustained, gradually declined over time. In the CuSi treatment, *HMA5* expression showed a similar downward trend but consistently remained lower than in the Cu treatment. Similarly, Cu strongly activated *NRAMP1* transcription, producing a sharp spike within 8 h and reaching levels well above the control (Figure 4I), thereby identifying *NRAMP1* as a Cu-responsive gene. Following this initial surge, expression gradually declined but remained elevated relative to the control. Interestingly, in the CuSi treatment, Si appeared to mitigate Cu’s impact, significantly dampening the early spike. Nevertheless, from day 3 to day 5, expression remained higher than in the Cu treatment. Si treatment increased *NRAMP1* expression only on day 3. Our results showed that under Cu excess, *YSL3* expression increased continuously (Figure 4J), with a marked rise at 8 h and a sharp peak by day 5. Si alone induced a gradual increase of *YSL3* that peaked on day 3, and then declined. In the combined CuSi treatment, *YSL3* expression followed a contrasting trend, decreasing over time and approaching control levels by day 3.

### 2.5. miRNAome Analysis

Exploratory miRNA profiling identified 87 candidate Cu- and/or Si-responsive miRNAs in cucumber roots, including 62 known and 25 candidate novel miRNAs (Figure S4). The key structural and biogenic characteristics of the identified novel miRNAs were determined using a custom bioinformatics pipeline (executed by Novogene). For the 25 novel miRNAs, a systematic filtering protocol was applied. Candidate sequences were strictly selected based on the presence of a well-defined stem-loop hairpin structure, a stable minimum free energy (MFE), and characteristic Dicer cleavage patterns. The comprehensive summary table (Table S7) shows minimum free energies (MFE, kcal/mol) as an indicator of secondary structure stability parameters for each novel miRNA.

**Figure 5:**
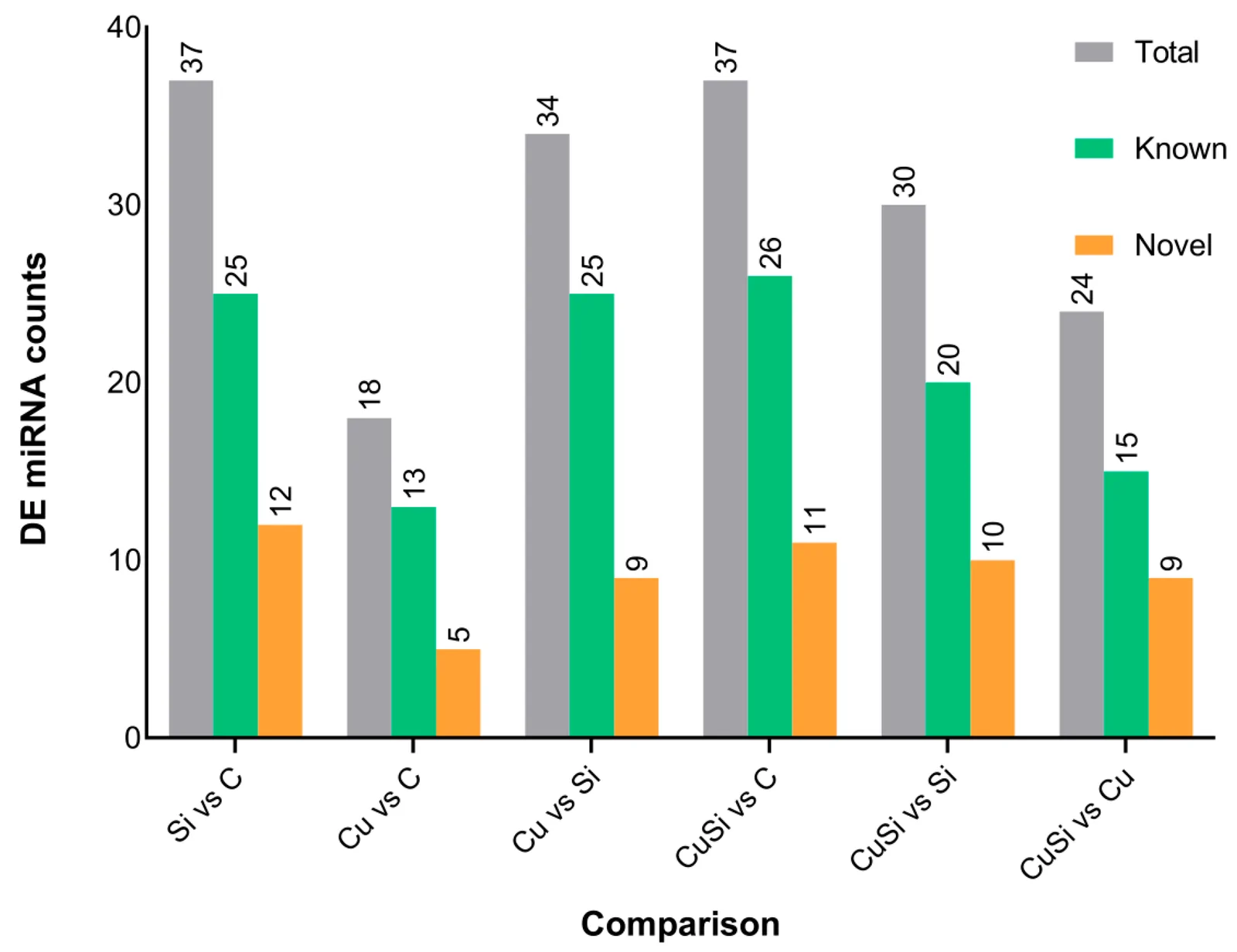
Distribution of candidate differentially expressed microRNAs (DE miRNAs) across pairwise treatment comparisons. Plants were grown under optimal Cu concentrations (C) or treated with 1.5 mM silicon (Si), 10 µM Cu (Cu), or co-treated with 10 µM Cu and 1.5 mM Si (CuSi). Samples were analyzed after five days of treatment.

**Figure 6:**
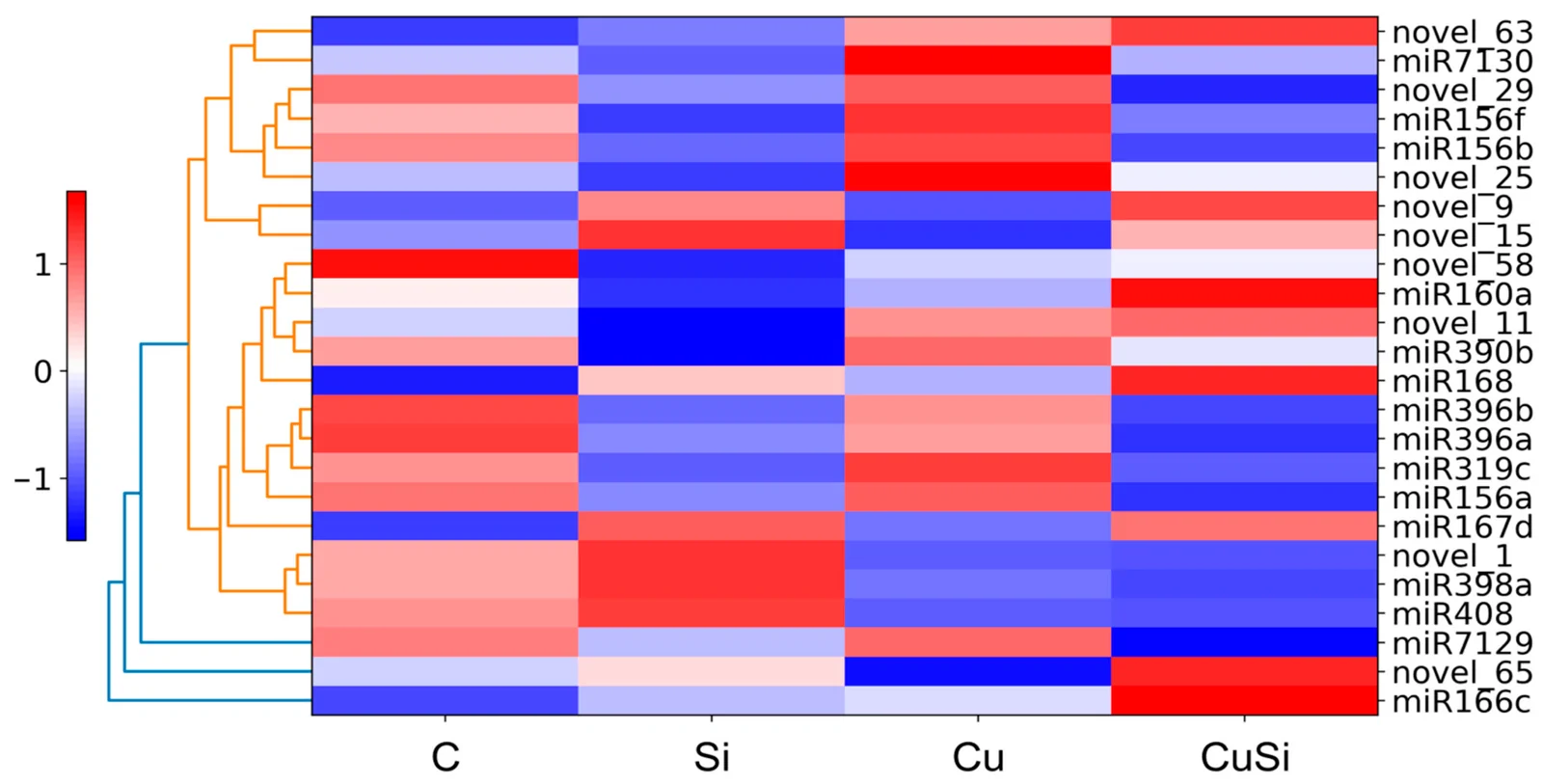
Heatmap showing the expression profiles of candidate known and candidate novel miRNAs differentially expressed between CuSi and Cu treatments. Plants were grown under optimal Cu concentrations (C) or treated with 1.5 mM silicon (Si), 10 µM Cu (Cu), or co-treated with 10 µM Cu and 1.5 mM Si (CuSi). Samples were analyzed after five days of treatment.

Candidate miRNAs exhibiting treatment-associated expression changes were detected across all pairwise comparisons, with the largest numbers observed in the Si vs. control and CuSi vs. control comparisons (Figure 5). These candidates included highly conserved miRNAs implicated in abiotic stress responses (e.g., miR166 and miR168), growth and development (miR319, miR396, and miR390), and Cu homeostasis (miR398 and miR408) (Figure 6). Selected candidate miRNAs were subsequently validated by qRT-PCR.

### 2.6. Validation of miRNA Expression by qRT-PCR

**Figure 7:**
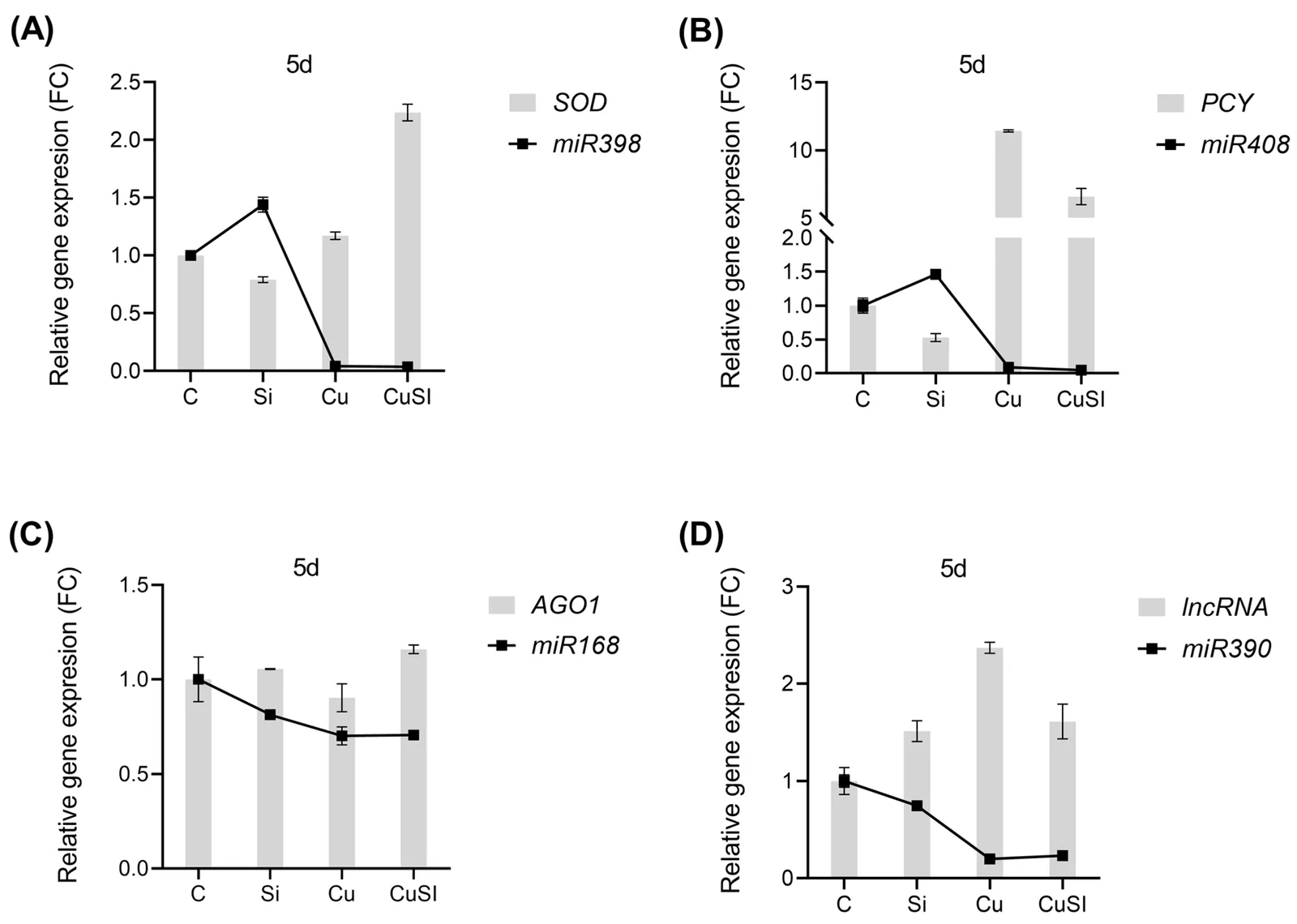
Relative expression of miRNAs and their predicted target genes in cucumber roots after 5 days (5 d) of Cu and/or Si treatment: miR398 and SOD (**A**), miR408 and PCY (**B**), miR168 and AGO1 (**C**), miR390 and lncRNA (**D**). The ordinate represents relative gene expression as a fold change. Data are presented as the mean ± SD (standard deviation, indicated by error bars) of values obtained in repeated experiments (*n* = 3). For statistical analysis (Table S5), two-way ANOVA followed by Tukey’s multiple comparison test was performed using GraphPad Prism 8.0. FC and SD values for all treatments are provided in Supplementary Table S6.

To validate the differential expression of miRNAs identified by miRNA sequencing, qRT-PCR assays were conducted to analyze miRNA expression levels in cucumber roots under different treatment conditions at four time points (8 h, 1 d, 3 d, and 5 d). Based on the screening miRNAome data, four miRNAs (miR398, miR408, miR168, and miR390), involved in various plant processes, were selected for validation and further analysis. qRT-PCR analysis showed that the presence of Cu caused both miR398 and miR408 expression levels to decline as early as 8 h after treatment initiation (Figure S5), and their expression continued to decrease until day 5, with lower levels observed in CuSi treatment than in Cu treatment (Figure 7). Similarly, miR390 expression declined throughout the experiment in both Cu and CuSi treatments (Figures S5–S7), reaching a lower level in the Cu treatment than in the CuSi treatment on day 5 (Figure 7). In contrast, miR168 exhibited a dynamic expression pattern during the time-course experiment, showing both upregulation (at 8 h in CuSi treatment and at day 3 in Cu treatment) and downregulation (on days 1 and 5 in both treatments) (Figures S5–S7 and Figure 7).

### 2.7. Prediction of miRNA Target Genes

The predicted target genes of the four selected miRNAs were predicted using psRobot and identified in the transcriptome data. The predicted target for miR398 was only the Cu/Zn superoxide dismutase (SOD). miR408 was predicted to have three targets with the same name—basic blue protein-like—which were identified as plantacyanin (PCY), based on Arabidopsis orthologs. miR168 was predicted to target three genes: argonaute 1 (AGO1) and two transcript variants of the GDP-mannose transporter GONST3. miR390 exhibited six predicted targets, including two long non-coding RNAs (lncRNAs)—uncharacterized transcript variants X1 and X2 (LOC105435804)—and four mRNAs: leucine-rich repeat receptor-like tyrosine-protein kinase PXC3 (which showed no expression changes in the transcriptome), kinesin-like protein KIN-7N, U-box domain-containing protein 52, and another uncharacterized mRNA (also showing no expression changes).

### 2.8. Verification of Predicted Target Gene Expression

To further validate the treatment-associated expression patterns of the candidate target genes identified from the exploratory transcriptome data, qRT-PCR was performed. The expression profiles of the predicted target transcripts in cucumber roots treated with Cu and/or Si at the different time points (8 h, 1 d, 3 d) are presented in Figures S5–S7. On day 5, Cu stress led to marked changes in the expression of both miR398 and its target gene SOD (Figure 7). In Cu-treated plants, *SOD* transcripts accumulated to levels slightly exceeding those observed in the control plants. The addition of Si (CuSi treatment) further increased *SOD* expression, reaching 2.2-fold compared with the control. In contrast, miR398 expression remained strongly suppressed in both Cu-containing treatments. The downregulation of *SOD* detected in the Si treatment corresponded to the inverse expression pattern between miR398 and its predicted target SOD, with similar fold-change values.

After 5 days of treatment, Cu exposure significantly affected the expression of both miR408 and its predicted target gene PCY (Figure 7). *PCY* transcript abundance increased approximately 11-fold in Cu-treated plants compared with the control. Interestingly, miR408 expression was downregulated by exactly 11-fold in the Cu treatment, showing an inverse expression pattern relative to the predicted target PCY. In CuSi-treated plants, the 6.5-fold upregulation of *PCY* was consistent with the downregulation of miR408. An inverse expression pattern was also observed under Si treatment, with *PCY* expression decreasing by approximately 0.5-fold and miR408 expression increasing by approximately 1.5-fold.

The expression pattern of *AGO1* was opposite among Cu-containing treatments after 5 days (Figure 7). *AGO1* transcript levels were slightly decreased in Cu-treated plants compared with the control, whereas Cu with Si supplementation led to a moderate increase in *AGO1* expression. Conversely, miR168 expression was lower in both Cu and CuSi treatments than in control plants.

The treatment-associated expression patterns of the lncRNA and miR390 exhibited a pronounced inverse expression pattern after 5 days of treatment. *lncRNA* expression increased across all analyzed treatments, reaching its highest level in Cu-treated plants (2.4-fold). The addition of Si under Cu stress attenuated this response, resulting in a 1.6-fold increase, while Si treatment alone resulted in a 1.5-fold increase in *lncRNA* expression. In contrast, miR390 expression decreased in all treatments, mirroring the changes observed for the *lncRNA* (Figure 7).

## 3. Discussion

Cu toxicity leads to the massive accumulation of Cu in roots and disrupts the homeostasis of other micronutrients, resulting in oxidative stress and inhibition of plant growth. Si-mediated alleviation of Cu toxicity has been reported previously in *A. thaliana* [32], rice [33], tobacco [34], and cucumber [29,30]. The present study aimed to provide a comprehensive insight into transcriptomic and miRNAomic changes underlying cucumber responses to Si supplementation under Cu stress, with the objective of integrating these findings with previously published data.

### 3.1. Effect of Si in Optimal Growth Conditions

Although considered screening data, it is noteworthy that Si treatment induced substantial transcriptomic changes (approximately 1800 candidate DEGs), indicating active modulation of root gene expression even in the absence of stress (Figure 1). Interestingly, a similar number of DEGs following Si treatment has also been reported in cucumber leaves [35]. The observed responses in transporter expression (Figure 4) suggest that Si functions not merely as a passive structural component but also as a regulator of molecular processes involved in nutrient acquisition and transport. Such transcriptional adjustments may establish a physiological state that enhances the plant’s capacity to respond to subsequent environmental challenges. The upregulation of transporter genes indicates that silicon enhances plant capacity for micronutrient uptake, thereby preparing plants for periods of nutrient deprivation. Hernandez-Apaolaza (2022) [36] suggested that priming plants with Si could alleviate micronutrient deficiency symptoms. Despite the prevailing view that Si exerts only limited direct effects on plants under non-stress conditions [37], our results, at least, support species- or genotype-specific transcriptional effects of Si, as suggested by Zhu et al. (2019) [35].

### 3.2. Effect of Si Under Cu Stress

Cu stress causes severe physiological disturbance, forcing plants to undergo extensive transcriptomic reprogramming to cope with its adverse effects. Our screening results revealed fewer candidate DEGs in CuSi than in Cu plants relative to the control, suggesting that Si attenuates the transcriptional response to Cu stress (Figure 1). Similar effects of Si have been reported previously under Cd stress [38] and NaCl stress [35]. Moreover, Cu-resistant genotypes exhibit fewer DEGs under Cu excess than susceptible ones [39]. The present data suggest that Si amendment shifts the transcriptomic profile toward a less pronounced Cu stress response. Exploratory KEGG enrichment analysis suggested the possible involvement of phenylpropanoid biosynthesis in the stress response (Figure 3). Although Si is frequently associated with the activation of phenylpropanoid metabolism under adverse conditions [40], the present data suggest attenuation of Cu-induced stress responses rather than direct inhibition of phenylpropanoid metabolism. Cu stress is known to stimulate the synthesis of phenolic compounds and lignin as part of the plant defense strategy against oxidative damage [41]. Previously, we demonstrated that Si supplementation decreased *PAL1* (phenylalanine ammonia-lyase) expression, total phenolics accumulation, and lignification in Cu-exposed roots, while alleviating oxidative stress [30]. Since PAL catalyzes the first committed step of the phenylpropanoid pathway, lower *PAL1* expression suggests diminished metabolic flux toward phenolic and lignin biosynthesis, concomitant with the decreased requirement for stress-induced phenylpropanoid metabolism and cell wall reinforcement in Si-supplied plants.

Exploratory functional clustering of candidate differentially expressed genes indicated enrichment of transport-related GO terms (Table 1). Metal transporters are critical for regulating intracellular Cu levels by coordinating influx, efflux, and compartmentalization. Studies have demonstrated that individual transporter or chelator genes can significantly influence Cu tolerance and overall plant growth [42,43,44]. While research in model organisms such as rice and *A. thaliana* has made considerable advances in understanding these regulatory mechanisms at both transcriptional and post-transcriptional levels, little is currently known about these processes in cucumber [45]. Since transporters directly regulate cellular metal fluxes and are essential determinants of Cu toxicity and tolerance, their transcriptional regulation may provide important insights into the mechanisms underlying Si-mediated alleviation of Cu stress. In this study, we examined the effects of Cu stress and Si supplementation on the expression of key metal transporter genes, with particular emphasis on relating these changes to previously determined metal concentrations in cucumber [29].

Cu transmembrane transporters are regulated at the transcriptional and/or protein level, depending on the specific transporter, plant species, and organ [46]. COPT1, a plasma membrane Cu influx transporter, is negatively regulated by intracellular Cu levels [47,48,49]. Under Cu deficiency, *COPT1* is upregulated in *A. thaliana* and rice, while in response to Cu excess, its expression is repressed [50,51,52]. Herein, we report significant downregulation of *COPT1* in cucumber roots grown under excess Cu treatment (Figure 4). This was accompanied by a marked decrease in *FRO4* expression, whose product reduces Cu^2+^ to Cu^+^ for COPT1-mediated uptake. While increased expression of this enzyme has previously been shown in *A. thaliana* roots under Cu deficiency [53], to our knowledge, this is the first report indicating transcriptional regulation of *FRO4* under Cu excess conditions. We also found a Cu-dependent downregulation of *ZIP2*, a ZIP family transporter responsible for Cu^2+^ acquisition at the root periphery [54].

Plants regulate cytosolic Cu by vacuolar sequestration. In rice, tonoplast-localized *COPT5* and *COPT7* are induced under Cu deficiency but repressed under 50 µM Cu excess [52], whereas *COPT5* expression in *A. thaliana* leaves is independent of Cu availability [50], highlighting species- and organ-specific regulation. In the present study, cucumber roots exhibited decreased *COPT5* expression, particularly under prolonged Cu exposure. In contrast, *HMA5*, a Cu efflux transporter, was upregulated (Figure 4). Although the subcellular localization of cucumber HMA5 is still elusive, homologs in *A. thaliana*, *Malus domestica* and *Silene vulgaris* localize to the plasma membrane and tonoplast, suggesting roles in Cu export from the root cells or vacuolar sequestration [50,52,55,56,57]. OsHMA5-mediated xylem loading promotes root-to-shoot Cu translocation in rice [58], whereas tonoplast-localized homologs, such as *S. vulgaris* SvHMA5I, may facilitate vacuolar Cu storage, thereby maintaining cytosolic Cu homeostasis while preventing excessive release, which can cause oxidative stress and damage the cell [42,56]. However, under severe or prolonged Cu stress, additional defense mechanisms, including cell wall immobilization, sequestration by metal chelators, and antioxidative protection, are also required [59].

During the early stages of Cu exposure in cucumber, Si amendment led to slight to moderate changes in the expression of Cu transporter genes, namely *COPT1*. During prolonged treatment, Si markedly mitigated the effects of Cu on *FRO4* and *COPT5* expression. These results suggest that Si-mediated stress alleviation is not primarily driven by transcriptional regulation of Cu transporters but rather by other mechanisms, described in our previous reports, including increased Cu-binding capacity of the root cell wall and accumulation of Cu-chelating compounds [29,30]. Similar findings are reported in studies of other research groups. For example, downregulation of rice *HMA* genes under both CuSi and CdSi treatments has been associated with decreased uptake of both metals, indicating that Si accumulation in roots restricts metal entry [33]. Likewise, in *A. thaliana*, Si attenuated Cu-induced *HMA5* expression, supporting the idea that Si mitigates Cu stress and reduces the need for detoxification responses [31]. On the contrary, *COPT1* was upregulated in Cu-treated *Nicotiana tabacum* roots, while Si decreased its expression, which correlated with Si-mediated stress alleviation and Cu uptake repression [34].

Beyond Cu-specific transport systems, excess Cu disrupts the homeostasis of other transition metals through shared uptake pathways and regulatory networks, impairing the acquisition of iron (Fe), Zn, and Mn and causing their deficiency in plants [2,3,29]. Crosstalk between Cu and Fe homeostasis is well documented and reflects both competition for transport systems and coordinated transcriptional regulation to maintain cellular redox balance [60]. Whereas Cu uptake is mainly handled by the FRO4/5–COPT1 pathway, FRO2 and IRT1 primarily function in Fe uptake but can also contribute to Cu acquisition [60,61]. Herein, we show that excess Cu repressed not only the primary Cu uptake system (*FRO4* and *COPT1*) but also *FRO2* and *IRT1* in cucumber roots (Figure 4), while Si downregulated Fe acquisition genes even more. Further research, including protein-level quantification, transporter activity assays, and functional studies employing a transgenic approach, will determine whether and to what extent changes in *FRO2* and *IRT1* contribute to limiting excessive Cu uptake. On the other hand, repression of the Fe transporter system likely contributed to the decreased Fe content in Cu-treated cucumber, as reported in our previous research [29]. Nevertheless, although Si further repressed Fe uptake genes, it did not cause a concomitant decrease in Fe content; instead, it ameliorated Fe deficiency in the leaves of Cu-treated cucumber, as shown in the same previous study by our team [29]. We also showed that this alleviating effect of Si is linked to increased Cu stress tolerance via the accumulation of metal-chelating compounds [29]. In contrast to the downregulation of *ZIP2*, which has the highest affinity for Cu, excess Cu strongly induced *ZIP4*, a major Zn transporter typically upregulated under Zn starvation. This result suggested a compensatory response to Cu-induced Zn depletion [6,62]. Similarly, *NRAMP1*, the primary high-affinity Mn transporter [7], was upregulated, likely reflecting a coping mechanism to alleviate secondary Mn deficiency. The induction of *ZIP4* and *NRAMP1* may therefore represent a compensatory response to Cu-induced Zn and Mn deficiencies, in an attempt to maintain homeostasis of these microelements. Silicon sustained higher *ZIP4* and *NRAMP1* transcript levels during prolonged Cu exposure (Figure 4), which correlates with increased Zn and Mn content in CuSi plants reported in our previous study [29].

Yellow Stripe-Like (YSL) transporters regulate the internal distribution of metal–nicotianamine complexes. Previous studies have shown that YSL3 facilitates Cu translocation [63], with mutants displaying decreased Cu accumulation in shoots [64], while overexpression increased leaf Cu levels [13]. Data on the expression of *YSL* genes in the roots of dicotyledonous plants under Cu excess remain scarce. In our study, Cu treatment increased *YSL3* expression, whereas Si amendment markedly decreased its transcript level (Figure 4). This finding is consistent with the decreased Cu translocation observed in CuSi-treated plants in our previous study [30].

The findings regarding Cu- and Si-induced modulation of gene expression presented herein require functional validation using proteomic studies and a transgenic approach to gain comprehensive insight into the complex network of genes involved in Cu uptake, translocation, and sequestration. Information on the regulation of their expression, localization, and function can be valuable for further applied research, including targeted manipulation of uptake or vacuolar/efflux transporters to engineer crops with reduced metal accumulation [65,66].

### 3.3. Si-Mediated Modulation of the Candidate miRNA-Target Expression in Cu-Stressed Cucumber

Cu toxicity induced substantial changes in miRNA-mediated regulatory networks. The inverse treatment-associated expression patterns observed between miRNAs and their predicted target genes are consistent with the canonical mechanism of post-transcriptional gene silencing mediated by plant miRNAs [67].

AGO1 is a central component of the RNA-induced silencing complex and is essential for miRNA-guided regulation of gene expression. Its expression is controlled through a conserved negative feedback loop involving miR168 [68]. The decreased expression of miR168 accompanied by increased *AGO1* transcript abundance in CuSi plants suggests that Si may contribute to maintaining the functionality of RNA silencing pathways during prolonged Cu exposure (Figure 7). Since AGO1 is required for the activity of numerous stress-responsive miRNAs, preservation of *AGO1* expression may facilitate the fine-tuning of adaptive responses under metal stress conditions. Similar stress-associated alterations in the miR168–*AGO1* regulatory circuit have been reported under various abiotic stresses, including drought, salinity, and heavy metal exposure [68].

miR408 is among the most conserved Cu-responsive miRNAs in plants and plays an important role in Cu homeostasis by regulating Cu-binding proteins and electron transport components [69]. Under Cu deficiency, miR408 is induced and promotes the degradation of transcripts encoding several Cu-containing proteins, including plantacyanin (PCY), thereby reallocating Cu to essential proteins involved in photosynthesis and respiration [70]. Herein, we observed the strong downregulation of miR408 in Cu-treated cucumber roots, along with the accumulation of *PCY* transcripts (Figure 7). Plantacyanin is a type I blue copper protein of the phytocyanin family that contains a conserved Cu-binding site and is thought to participate in electron transfer, redox regulation, and Cu homeostasis [71,72]. Recent functional evidence in *A. thaliana* further supports a role of the miR408–PCY module in regulating reactive oxygen species homeostasis and stress responses [20]. Thus, strong repression of miR408 accompanied by increased *PCY* transcript abundance in Cu-treated cucumber roots may represent a Cu-responsive adjustment of Cu-protein allocation and redox-associated processes. Nevertheless, in the present study, PCY should be considered a predicted miR408 target, and the observed inverse expression pattern does not, by itself, demonstrate direct miRNA-mediated regulation. The lower PCY transcript abundance observed in CuSi plants compared with Cu-treated plants suggests that Si modified this Cu-associated transcriptional response. However, transcript abundance alone cannot determine whether this change reflects altered Cu allocation, redox regulation, or another stress-responsive process. Similar observations have been reported in plants exposed to heavy metals, where Si decreased metal toxicity by limiting metal uptake, improving nutrient balance, and decreasing oxidative damage [40,71,72,73]. The continued suppression of miR408 in CuSi plants suggests that Cu exposure continued to influence this regulatory module despite Si supplementation.

Among the analyzed regulatory modules, the miR398–SOD pair displayed one of the strongest and most biologically coherent responses to Cu stress [74,75]. The pronounced increase in *SOD* transcript abundance is one of the clearest molecular responses identified in the present study and indicates activation of antioxidant defense under excess Cu. Importantly, Si further enhanced this response. In CuSi-treated plants, *SOD* expression reached approximately 2.2-fold the control level, exceeding the response observed under Cu treatment alone. Thus, unlike several Cu-responsive transporter genes whose expression was moderated by Si supplementation, the SOD response was strengthened in the presence of Si. This finding suggests that Si does not simply attenuate Cu-induced molecular responses but can selectively enhance components of the plant defense system, particularly antioxidant protection. This interpretation is strongly supported by our previous study under the same Cu and Si concentrations, in which Si increased SOD activity and enhanced the accumulation of the Cu/Zn-SOD isoforms in cucumber roots [30,76]. The present transcript-level response therefore complements the previous protein and enzymatic data and further supports Cu/Zn-SOD as an important component of the Si-associated response to excess Cu, consistent with the recognized Si-induced enhancement of plant antioxidant capacity under abiotic stress conditions [77]. The concomitant reduction in miR398 is also biologically relevant, as miR398 is a well-established component of plant Cu homeostasis and oxidative-stress responses and is associated with the regulation of Cu/Zn-SOD transcripts [74,78]. Although the miR398-Cu/Zn-SOD regulatory module was validated in several plant species, additional experimental evidence is required to confirm miR398-mediated regulation of the SOD transcript in cucumber and its role in Si-induced stress alleviation.

Long non-coding RNAs (lncRNAs) have emerged as important components of plant stress responses and may interact with miRNAs through several mechanisms, including direct targeting, target mimicry, or other RNA-mediated regulatory processes [79].

In the present study, Cu treatment led to a strong reduction in miR390 accompanied by increased transcript abundance of its predicted target *lncRNA*, resulting in an inverse expression pattern. The biological significance of this association is potentially relevant because miR390 is an evolutionarily conserved regulator of the TAS3–tasiARF pathway, which influences Auxin Response Factor (ARF) activity and root development [80]. Changes in miR390 abundance could therefore affect developmental and stress-responsive signaling pathways in roots.

**Figure 8:**
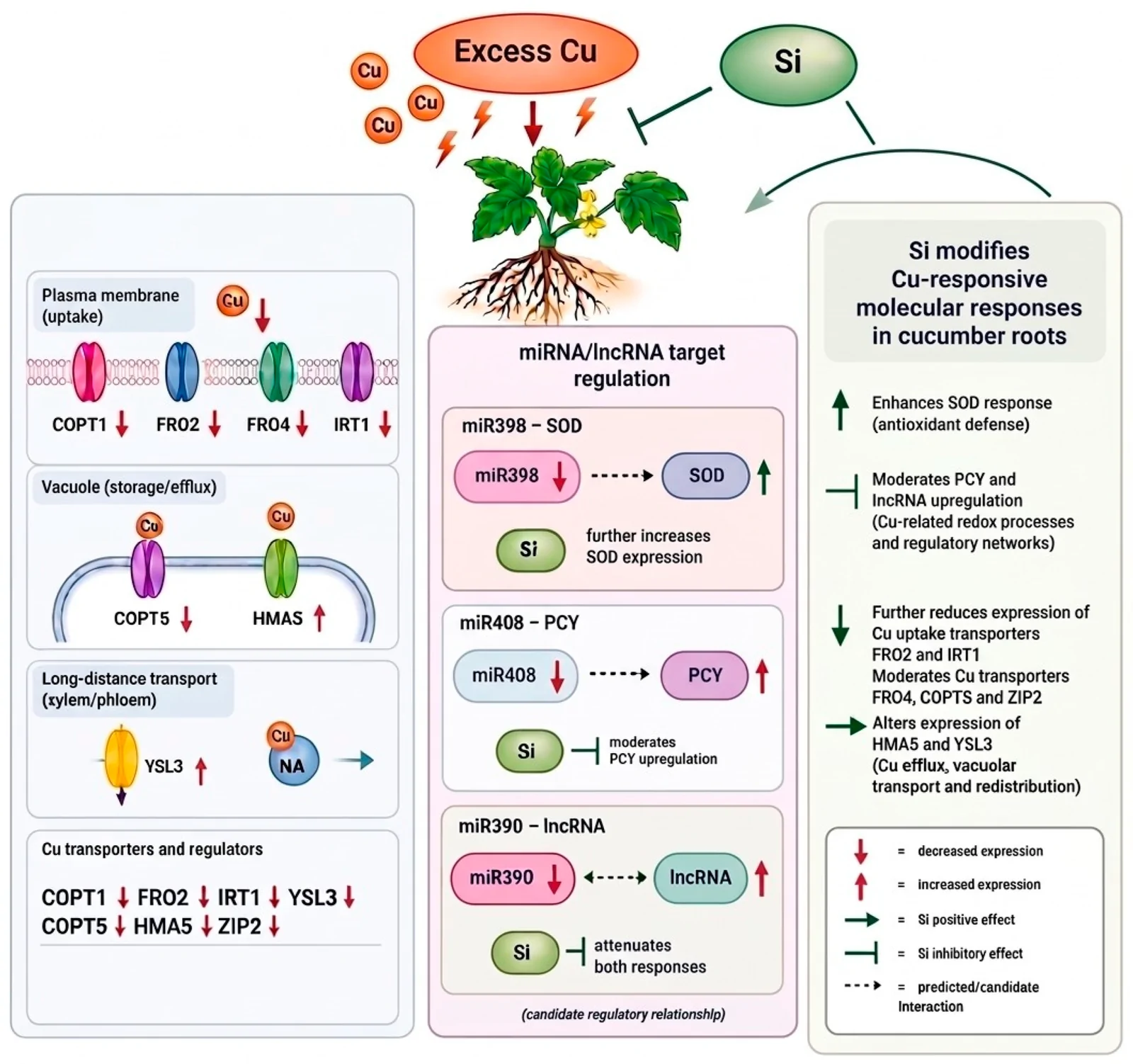
A diagram illustrating how Si mitigates the effects of excess Cu in cucumber plants, focusing on root molecular responses and the regulation of transporter expression. The upper center features a stressed cucumber plant subjected to excess Cu (Cu spheres), which is counteracted by Si (Si oval). Below the plant, a central panel represents the alternation of miRNA and predicted targets: Si further enhances the expression of SOD (antioxidant defense), while decreasing miR398; Si moderates the PCY/miR408 module (involved in redox processes); and Si attenuates miR390/lncRNA responses. The left column represents the modulation of specific Cu transporter expression, showing Si’s potential roles in decreasing expression of some transporters (e.g., FRO2 and IRT1), while moderating expression of others (e.g., COPT5 and FRO4). The right panel summarizes the cellular effects.

However, the present data do not establish whether the analyzed lncRNA is a direct miR390 target or whether it could function as a target mimic/decoy that affects miR390 availability. These mechanisms are conceptually distinct and cannot be discriminated on the basis of inverse treatment-associated expression patterns alone. Therefore, we refer to this association as a candidate miR390–lncRNA regulatory relationship. The attenuation of both Cu-associated miR390 and lncRNA responses in CuSi plants further suggests that Si modifies this expression relationship under Cu stress, but the molecular basis of this effect remains unknown. Experimental approaches such as degradome sequencing, 5′ RLM-RACE, reporter assays, or RNA interaction/pull-down experiments are needed to determine whether the lncRNA directly interacts with miR390 and to establish its functional significance. A mechanistic model summarizing our results is provided in Figure 8, depicting the pathways through which Si alleviates Cu toxicity by regulating root molecular responses and transporter expression.

Although the observed transcriptional changes are consistent with a role for Si in modulating gene regulatory processes during Cu stress, the present data do not establish direct mechanistic relationships. Further functional studies are required to determine whether these transcriptional responses are causally involved in Si-mediated alleviation of Cu stress.

## 4. Materials and Methods

### 4.1. Plant Cultivation and Treatments

Cucumber (*Cucumis sativus* L. cv. Chinese long) seeds were surface sterilized with sodium hypochlorite and soaked in a saturated calcium sulfate solution with aeration overnight. A hundred seeds were set to germinate in moist filter paper in the dark at 25 °C, and after four days the seedlings were transferred to hydroponic culture at the Institute of Molecular Genetics and Genetic Engineering, University of Belgrade (five plants per 3 L-plastic pot). Nutrient solution contained (in mM): 2.0 Ca(NO_3_)_2_, 0.7 K_2_SO_4_, 0.5 MgSO_4_, 0.1 KH_2_PO_4_, 0.1 KCl, and (in μM): 10 H_3_BO_3_, 0.5 ZnSO_4_, 0.5 MnSO_4_, 0.2 CuSO_4_, 0.01 (NH_4_)_6_Mo_7_O_24_, 20 Fe(III)EDTA. pH was monitored daily and remained at 6. Aeration was constantly provided, and the nutrient solution was renewed every two days. Photoperiod was 16/8 h, the temperature regime was 24/20 °C (light/dark), and photon flux density of 250 μmol m^2^ s^−1^ at plant height was provided by LED panels (Apollo 8, Cidly Co., Ltd., Shenzhen, China). After 7 days of preculture, the plants were separated into two groups: plants grown with 0.2 μM CuSO_4_ (control, C) and plants treated with 10 μM CuSO_4_ (Cu). In each group, 1.5 mM Si was added to half of the plants. Silicon was applied as freshly prepared Si(OH)_4_, obtained by passing Na_2_SiO_3_ through a cation-exchange resin (Amberlite IR-120, H^+^ form; Acros Organics, Geel, Belgium). In total, there were four groups of plants: control (0.2 μM CuSO_4_, -Si), Si plants (0.2 μM CuSO_4_, + 1.5 mM Si(OH)_4_), Cu plants (10 μM CuSO_4_, -Si), and CuSi plants (10 μM CuSO_4_, +1.5 mM Si(OH)_4_). Cu and Si concentrations were selected in the context of our previous cucumber Cu/Si experiments and were intended to provide a moderate Cu-stress condition under which the molecular response to Si supplementation could be examined [30]. The experiments consisted of three replicates (pots) having five plants per replicate. The transcriptome and small-RNA sequencing analyses were performed on root material collected after five days of treatment, using one pooled sample per treatment. Real-time PCR analyses were performed at 8 h, 1 day (1 d), 3 days (3 d), and 5 days (5 d) after the beginning of the treatments.

### 4.2. RNA Extraction

Root samples were collected, frozen in liquid nitrogen, and ground into a fine powder. Total RNA was extracted with buffer containing 8.0 M guanidine hydrochloride, 20 mM 2-(N-morpholino) ethanesulfonic acid (MES), 20 mM ethylenediaminetetraacetic acid (EDTA), and 2% β-mercaptoethanol as described by Trindade et al. 2010 [78]. After vortexing, an equal volume of phenol (pH 5.2) was added, and the samples were centrifuged at 12,000 RCF for 10 min. Supernatants were collected, and an equal volume of a mixture containing phenol: chloroform: isoamyl alcohol in a 25:24:1 ratio was added. After centrifugation at 12,000 RCF for 2 min, the last step was repeated. The upper phase containing nucleic acids was collected, and two volumes of absolute ethanol and 1/20 volume of 4 M sodium acetate (pH 5.2) were added. After incubation at −20 °C overnight, the precipitated RNA was pelleted at 12,000 RCF for 10 min. The RNA pellets were washed with 70% ethanol and then dissolved in RNase-free water. RNA samples were kept at −80 °C.

A DNA-free kit for DNase treatment (Thermo Fisher Scientific™, Waltham, MA, USA) was used according to the manufacturer’s protocol to remove DNA from the RNA extracts.

### 4.3. mRNA Sequencing and Bioinformatics Analysis

Messenger RNA (mRNA) was purified using poly-T oligo-attached magnetic beads from total RNA extracted from cucumber plant roots treated for five days as described. RNA integrity and purity were assessed using an Agilent 2100 Bioanalyzer (Agilent Technologies, Santa Clara, CA, USA). Sequencing libraries were prepared following poly(A) enrichment and cDNA synthesis and subsequently sequenced on the Illumina NovaSeq X Plus platform (Novogene Bioinformatics Technology Co., Ltd., Cambridge, UK) using a paired-end 150 bp (PE150) sequencing strategy. After quality filtering, clean paired-end reads were aligned to the cucumber reference genome (NCBI assembly: *Cucumis sativus* GCF_000004075.3_Cucumber_9930_v3) using HISAT2 v2.0.5 [81]. Gene-level read counts were generated with FeatureCounts v1.5.0-p3 [82]. Prior to differential expression analysis, read counts were adjusted by a scaling normalization factor in the edgeR package [83]. Candidate differentially expressed genes (DEGs) were identified using the edgeR R package (v3.22.5). Genes with an adjusted *p*-value (padj) < 0.05 and an absolute log2 fold change (|log2FC|) > 1 were considered significantly differentially expressed. Gene Ontology (GO) enrichment analysis of candidate DEGs was performed using the clusterProfiler R package. GO terms with a corrected *p*-value < 0.05 were considered significantly enriched, and the 15 most significantly enriched GO terms were selected for presentation [84]. Kyoto Encyclopedia of Genes and Genomes (KEGG) pathway enrichment analysis was also conducted using the clusterProfiler package to identify significantly enriched metabolic and signaling pathways [85]. Overviews of the sequencing and mapping statistics are provided in Table S1. Since mRNA sequencing was performed without biological replicates, the transcriptomic results should be considered as screening data. Consequently, the identified candidate DEGs and enrichment analyses were used primarily to generate hypotheses and guide subsequent validation experiments.

Within the transcript pool, lncRNAs were classified according to the established Novogene lncRNA-identification pipeline, which also includes mRNA in its analysis. Therefore, the identified lncRNAs represent the polyadenylated lncRNA fraction primarily and should not be considered a complete representation of the cucumber lncRNA population.

The standard lncRNA sequencing pipeline of eukaryotic species comprised the following steps: The first step was ensuring that the raw data met the quality control standards. The raw data were checked for error rate distribution and GC content, and passed through data filtering. The RNA molecules were then mapped to the reference genome (ncbi_cucumis_sativus_gcf_000004075_3_cucumber_9930_v3), and structural variations were then analyzed. The screened transcripts were classified into two groups: known and novel transcripts. The lncRNA identification pipeline included discarding transcripts < 200 nucleotides in length to eliminate miRNA, rRNA, tRNA, snoRNA, siRNA, and eliminating transcripts classified as coding.

### 4.4. Small RNA Sequencing and Bioinformatics Analysis

Total RNA from samples treated for 5 days, as described, was used as the input material for small RNA library preparation. Following ligation of adapters to the 3′ and 5′ ends of small RNAs, first-strand cDNA synthesis was performed using a reverse transcription primer. Double-stranded cDNA libraries were subsequently generated by PCR amplification. After purification and size selection, libraries containing inserts of 18–40 nt were selected for sequencing. Small RNA libraries were sequenced on an Illumina NovaSeq 6000 platform (Novogene Bioinformatics Technology Co., Ltd., Cambridge, UK) using a single-end 50 bp sequencing strategy. After quality control and adapter trimming, clean reads were mapped to the reference genome using Bowtie [86]. To characterize small RNA expression and genomic distribution, alignments were performed allowing either no mismatches or up to one mismatch. Small RNA sequences identified as microRNAs (miRNAs) were subjected to further analyses. Known miRNAs were identified by comparison against miRBase release 22.0. Because only a limited number of *C. sativus* miRNAs are currently deposited in miRBase, *Cucumis melo* miRNA annotations were additionally used as a reference for homology-based identification. Candidate novel miRNAs were predicted using the integrated tools miREvo [87] and miRDeep2 [88], which evaluate characteristic miRNA precursor features, including secondary structure, Dicer cleavage sites, and minimum free energy of folding from unannotated small RNA sequences. Potential target genes of identified miRNAs were predicted using the psRobot_tar module implemented in psRobot [89]. Target prediction was performed using the psRobot_tar module, which evaluates complementarity across the full length of the miRNA-target duplex using a modified Smith–Waterman alignment algorithm [89]. Consistent with the plant miRNA targeting paradigm, particular attention was given to the absence of mismatches in the central region of the duplex (approximately nucleotides 10–11 of the mature miRNA), which corresponds to the predicted AGO1-mediated cleavage site. miRNA expression levels were quantified as transcripts per million (TPM) according to the following formula [90]: TPM = (mapped read count/total mapped reads) × 10^6^. Prior to differential expression analysis, read counts were normalized using the trimmed mean of M-values (TMM) method. Differential expression analysis was subsequently performed using the standard Novogene analysis pipeline. Hierarchical clustering and heatmap visualization of selected differentially expressed miRNAs were performed using Heatmapper. Since biological replicates were not included, the resulting small RNA expression profiles were considered exploratory and were used primarily for candidate miRNA identification. Key expression patterns were subsequently validated by RT-qPCR.

### 4.5. Primer Design

The sequences of miRNA398, miRNA408, miR168 and miR390 were obtained from the miRBase sequence database. Stem-loop primers for miRNA reverse transcription (RTmiR398: 5′-gtc gta tcc agt gca ggg tcc gag gta ttc gca ctg gat acg acc agg gg-3′; RTmiR408: 5′-gtc gta tcc agt gca ggg tcc gag gta ttc gca ctg gat acg acg cca gg-3′; RTmiR168: 5′-gtc gta tcc agt gca ggg tcc gag gta ttc gca ctg gat acg act ccc ga-3′; RTmiR390: 5′-gtc gta tcc agt gca ggg tcc gag gta ttc gca ctg gat acg acg gcg ct-3′) were designed according to Varkonyi-Gasic et al. (2007) [91]. Additional forward primers specific to the miRNA sequences were designed to match the miRNA398, miRNA408, miR168, and miR390 sequences but without the last 6 nucleotides at the 3′ end of the corresponding miRNAs. A universal primer corresponding to the universal backbone of the stem-loop primer was used as the reverse primer in further amplification. Its sequence was obtained from Varkonyi-Gasic et al. (2007) [91]. Sequences for *COPT1*, *FRO4*, *IRT1*, *FRO2*, *ZIP2*, *ZIP4*, *COPT5*, *HMA5*, *NRAMP1*, *YSL3*, *AGO1*, *SOD*, and *PCY* were obtained from the NCBI (National Center for Biotechnology Information) database, and their accession numbers are listed in Table S2. Additionally, three pairs of primers for house-keeping gene amplification were designed. One pair was specific to actin, and another to F-box, while the U6 spliceosomal RNA gene was used as a reference gene for miRNA expression analyses. All primers used in this study are listed in Table S2.

### 4.6. Reverse Transcription

Reverse transcription (RT) reactions were done using miRNA-specific stem-loop primers or Random Hexamer Primers. Reverse transcriptions were performed in a total volume of 20 µL starting with 1 µg of total RNA and 1 μM of stem-loop RT primer specific for miR398, miR408, miR168, or miR390 in four separate reactions. Primer denaturation was performed for 5 min at 65 °C. Further, reactions were incubated on ice for 2 min. Master mix was prepared by adding 4 µL 5X Reaction buffer (Thermo Fisher Scientific™, Waltham, MA, USA), 2 µL 10 mM dNTP (Thermo Fisher Scientific™, Waltham, MA, USA), 0.5 µL RiboLock RNase Inhibitor (Thermo Fisher Scientific™, Waltham, MA, USA), and 1 µL RevertAid Reverse Transcriptase (Thermo Fisher Scientific™, Waltham, MA, USA) to each reaction. After incubation for 10 min at 25 °C, reverse transcription was performed at 43 °C for 1 h. Final transcriptase deactivation was performed at 70 °C for 10 min.

In parallel, exactly the same amount of RNA was reverse-transcribed under the same conditions as previously, using Random Hexamer Primers (Thermo Fisher Scientific™, Waltham, MA, USA) as RT primers for subsequent analyses of transporters and predicted miRNA target genes.

### 4.7. Quantitative Real-Time PCR

Quantitative PCR was performed with Power SYBR™ Green qPCR Master Mix (Thermo Fisher Scientific™, Waltham, MA, USA) and 400 nM of each primer. Each PCR reaction was done in triplicate, including no-template controls. Quantitative PCR was conducted on the Magnetic Induction Cycler (Mic qPCR, Bio Molecular Systems, Brisbane, Queensland, Australia) using the following program parameters: 10 min at 95 °C, followed by 40 cycles of 15 s at 95 °C and 1 min at 60 °C. Results were analyzed with MicPCR software v2.12.7 (Bio Molecular Systems, Brisbane, Queensland, Australia) and presented as relative quantification, normalized to a reference gene and control, and to untreated samples, i.e., using the 2^−ΔCt^ method. qPCR analysis was performed using three biological replicates, with each sample run in three technical replicates. Data normalization was achieved using two endogenous reference genes. Reference genes for expression normalization were selected based on their stability under heavy metal stress, as previously evaluated by [92]. We selected two reference genes for our normalization: F-box and actin. The relative quantification of all target transcripts was calculated against the geometric mean of both reference genes, which was integrated into the final statistical analysis. For the normalization of microRNA (miRNA) expression levels, U6 small nuclear RNA (snRNA) was employed as a reliable endogenous control, following the methodology described by [93]. Primer amplification efficiencies were systematically evaluated and ranged between 80% and 100%. Single peaks in the melting-curve analysis confirmed the specificity of all amplifications. Furthermore, the efficiencies of the target and reference genes were verified to be sufficiently similar, thereby justifying the application of the 2^−ΔCt^ method.

### 4.8. Statistical Analysis

For statistical analysis, two-way ANOVA followed by Tukey’s multiple comparison test with a *p*-value of <0.05 considered statistically significant, was performed using GraphPad Prism 8.0. The two independent factors evaluated in the model were treatment (Control, Cu, Si, and CuSi) and Time (8 h, 1 d, 3 d and 5 d). Prior to performing the ANOVA, data normality and homogeneity of variance were verified using the Shapiro–Wilk and Brown-Forsythe tests, respectively. Although the full two-way model was applied, data representation and discussion focus primarily on the treatment factor as the main biological driver. Pairwise comparisons among the groups were conducted using Tukey’s multiple comparisons test. Differences that reached a *p*-value of less than 0.05 were considered statistically significant: * *p* < 0.05, ** *p* < 0.01, *** *p* < 0.001, **** *p* < 0.0001, ns—not significant.

## 5. Conclusions

Overall, our findings demonstrate that Si modulates both coding and non-coding RNAs under optimal and Cu-stress conditions, indicating that post-transcriptional regulation constitutes an additional layer of Si’s effect on plants. Si moderated most of the gene expression changes induced by Cu treatment, suggesting that transcript-level alterations are not the primary mechanism by which this beneficial element exerts its effects in cucumber roots. Rather, as demonstrated in our previous studies, Si appears to alleviate Cu excess in cucumber by reducing metal availability, enhancing Cu immobilization and detoxification, and improving antioxidant capacity. Notably, Si supplementation amplified the Cu-induced upregulation of *Cu/Zn SOD* transcript levels, concomitant with the downregulation of miR398, a well-established negative regulator of *SOD* transcripts in plants. Our earlier research identified enhanced antioxidative protection, including increased SOD protein abundance and enzymatic activity, as a key mode of Si action. The present results further reveal that Si modulates *Cu/Zn SOD* expression at the transcriptional level, underscoring the critical role of this antioxidative enzyme in Si-mediated stress mitigation. Nonetheless, additional experimental validation is required to confirm the involvement of miR398 in regulating *SOD* transcripts in cucumber and its contribution to Si-induced stress alleviation. Future investigations that integrate physiological and biochemical aspects of Si action with its effects on post-transcriptional regulatory mechanisms will advance our understanding of the molecular basis of Si-mediated heavy metal tolerance.

## Figures and Tables

**Table 1:** Top 15 Gene Ontology (GO) terms identified by exploratory enrichment analysis of candidate differentially expressed genes (DEGs) in response to Cu and/or Si treatments, grouped into the three main GO categories: biological process (BP), molecular function (MF), and cellular component (CC).

Category	GO ID	GO Term	Si vs. C	Cu vs. C	Cu vs. Si	CuSi vs. C	CuSi vs. Si	CuSi vs. Cu
BP	GO:0006979	response to oxidative stress		54 (29↑ 25↓)	52 (23↑ 29↓)	48 (27↑ 21↓)	42 (20↑ 22↓)	24 (13↑ 11↓)
	GO:0050896	response to stimulus		203 (117↑ 86↓)	179 (85↑ 94↓)	175 (107↑ 68↓)	132 (64↑ 68↓)	
	GO:0006950	response to stress		110 (60↑ 50↓)	92 (40↑ 52↓)	88 (49↑ 39↓)	68 (30↑ 38↓)	
	GO:0005975	carbohydrate metabolic process		194 (98↑, 96↓)	167 (81↑ 86↓)	156 (85↑ 71↓)	121 (61↑ 60↓)	
	GO:0071554	cell wall organization or biogenesis		37 (20↑ 17↓)	36 (17↑ 19↓)	35 (20↑ 15↓)	30 (12↑ 18↓)	
CC	GO:0005576	extracellular region		30 (13↑ 17↓)	23 (10↑ 13↓)	24 (7↑ 17↓)	17 (5↑ 12↓)	16 (7↑ 9↓)
	GO:0048046	apoplast		19 (5↑ 14↓)	14 (4↑ 10↓)	19 (4↑ 15↓)	12 (3↑ 9↓)	10 (6↑ 4↓)
MF	GO:0020037	heme binding	37 (28↑ 9↓)	174 (96↑ 78↓)	172 (84↑ 88↓)	150 (94↑ 56↓)	123 (65↑ 58↓)	84 (39↑ 45↓)
	GO:0016209	antioxidant activity		61 (33↑ 28↓)	59 (27↑ 32↓)	53 (30↑ 23↓)	46 (23↑ 23↓)	28 (15↑ 13↓)
	GO:0003700	DNA binding transcription factor activity	42 (32↑ 10↓)	177 (144↑, 33↓)	152 (118↑ 34↓)	128 (101↑ 27↓)	102 (77↑ 25↓)	63 (15↑ 48↓)
	GO:0022857	transmembrane transporter activity		218 (120↑, 98↓)	189 (100↑ 89↓)	165 (105↑, 60↓)	140 (85↑ 55↓)	82 (53↑ 29↓)
	GO:0015267	channel activity		49 (24↑ 25↓)	42 (18↑ 24↓)	39 (20↑ 19↓)	32 (13↑ 19↓)	
	GO:0005507	copper ion binding			23 (3↑ 20↓)	21 (6↑ 15↓)	17 (4↑ 13↓)	
	GO:0015297	antiporter activity		36 (25↑ 11↓)	32 (20↑ 12↓)	29 (23↑ 6↓)	24 (17↑ 7↓)	
	GO:0022804	active transmembrane transporter activity			47 (28↑ 19↓)		36 (25↑ 11↓)	

## Data Availability

The datasets generated during the current study are available in the NCBI Sequence Read Archive (SRA). The data discussed in this publication have been deposited in the NCBI Gene Expression Omnibus and can be accessed through GEO Series accession number GSE345100 at: https://www.ncbi.nlm.nih.gov/geo/query/acc.cgi?acc=GSE345100 (accessed on 31 July 2026).

## Supplementary Materials

The following supporting information can be downloaded at: https://www.mdpi.com/article/10.3390/plants15203189/s1, Figure S1. Venn diagram illustrating candidate genes that are uniquely and commonly differentially expressed across treatments. Figure S2. Cluster analysis grouped candidate differentially expressed genes (DEGs) with similar treatment-associated expression patterns into four subclusters, based on the log_2_ fold changes in their expression levels relative to the control group. Figure S3. Transcript Size Distributions of candidate lncRNAs found in cucumber roots. Figure S4. Venn diagram illustrating candidate miRNAs that are uniquely or commonly differentially expressed across treatments. Figure S5. Relative expression of microRNAs and their predicted target genes in cucumber roots after 8 h of Cu and/or Si treatment. Figure S6. Relative expression of microRNAs and their predicted target genes in cucumber roots after 1 day (1 d) of Cu and/or Si treatment. Figure S7. Relative expression of microRNAs and their predicted target genes in cucumber roots after 3 days (3 d) of Cu and/or Si treatment. Table S1. Summary of data production. Table S2. List of primers used in the study. Table S3. Statistical analysis of relative gene expression of transporters under Si, Cu, and CuSi treatments. Table S4. Relative gene expression levels of metal transporters in cucumber roots under Cu and/or Si treatments presented as log_10_ mean fold change (FC) and propagated standard deviation (SD) derived from the original sample variance using the logarithmic error propagation formula. Table S5. Statistical analysis of relative gene expression of miRNAs and their predicted target genes under Si, Cu, and CuSi treatments. Table S6. Relative expression of miRNAs and their predicted target genes in cucumber roots presented as mean FC and SD. Table S7. Sequence characteristics and energy profiles of predicted novel miRNAs.

## Abbreviations

The following abbreviations are used in this manuscript:

COPT	Copper transporter
FRO	Ferric reductase oxidase
ZIP	Zrt/Irt-like protein
IRT	Iron-regulated transporter
NRAMP	Natural resistance-associated macrophage proteins
HMA	Heavy metal ATPase
YSL	Yellow stripe-like
NA	nicotianamine
SPL	SQUAMOSA promoter binding protein-like
lncRNAs	long non-coding RNAs
miRNAs	microRNAs
AGO	Argonaute
Cu/Zn SOD	Copper/zinc superoxide dismutase
PCY	Plantacyanin
DEGs	differentially expressed genes
GO	Gene ontology
KEGG	Kyoto encyclopedia of genes and genomes
PAL	Phenylalanine ammonia-lyase

## References

[1] Marschner P. (2012). Marschner’s Mineral Nutrition of Higher Plants.

[2] Xu E., Liu Y., Gu D., Zhan X., Li J., Zhou K., Zhang P., Zou Y. (2024). Molecular Mechanisms of Plant Responses to Copper: From Deficiency to Excess. Int. J. Mol. Sci..

[3] Chen G., Li J., Han H., Du R., Wang X. (2022). Physiological and Molecular Mechanisms of Plant Responses to Copper Stress. Int. J. Mol. Sci..

[4] Puig S., Lee J., Lau M., Thiele D.J. (2002). Biochemical and genetic analyses of yeast and human high affinity copper transporters suggest a conserved mechanism for copper uptake. J. Biol. Chem..

[5] Sancenón V., Puig S., Mateu-Andrés I., Dorcey E., Thiele D.J., Peñarrubia L. (2004). The Arabidopsis copper transporter COPT1 functions in root elongation and pollen development. J. Biol. Chem..

[6] Wintz H., Fox T., Wu Y.-Y., Feng V., Chen W., Chang H.-S., Zhu T., Vulpe C. (2003). Expression profiles of *Arabidopsis thaliana* in mineral deficiencies reveal novel transporters involved in metal homeostasis. J. Biol. Chem..

[7] Cailliatte R., Schikora A., Briat J.-F., Mari S., Curie C. (2010). High-affinity manganese uptake by the metal transporter NRAMP1 is essential for Arabidopsis growth in low manganese conditions. Plant Cell.

[8] Vert G., Grotz N., Dédaldéchamp F., Gaymard F., Guerinot M.L., Briat J.-F., Curie C. (2002). IRT1, an Arabidopsis transporter essential for iron uptake from the soil and for plant growth. Plant Cell.

[9] Puig S. (2014). Function and regulation of the plant COPT family of high-affinity copper transport proteins. Adv. Bot..

[10] Klaumann S., Nickolaus S.D., Fürst S.H., Starck S., Schneider S., Ekkehard Neuhaus H., Trentmann O. (2011). The tonoplast copper transporter COPT5 acts as an exporter and is required for interorgan allocation of copper in *Arabidopsis thaliana*. New Phytol..

[11] Garcia-Molina A., Andrés-Colás N., Perea-García A., del Valle-Tascón S., Peñarrubia L., Puig S. (2011). The intracellular Arabidopsis COPT5 transport protein is required for photosynthetic electron transport under severe copper deficiency. Plant J..

[12] Mijovilovich A., Leitenmaier B., Meyer-Klaucke W., Kroneck P.M.H., Götz B., Küpper H. (2009). Complexation and toxicity of copper in higher plants. II. Different mechanisms for copper versus cadmium detoxification in the copper-sensitive cadmium/zinc hyperaccumulator *Thlaspi caerulescens* (Ganges ecotype). Plant Physiol..

[13] Chu H.-H., Chiecko J., Punshon T., Lanzirotti A., Lahner B., Salt D.E., Walker E.L. (2010). Successful reproduction requires the function of Arabidopsis Yellow Stripe-Like1 and Yellow Stripe-Like3 metal-nicotianamine transporters in both vegetative and reproductive structures. Plant Physiol..

[14] Yamasaki H., Hayashi M., Fukazawa M., Kobayashi Y., Shikanai T. (2009). SQUAMOSA promoter binding protein–like7 is a central regulator for copper homeostasis in Arabidopsis. Plant Cell.

[15] Yu Y., Zhang Y., Chen X., Chen Y. (2019). Plant Noncoding RNAs: Hidden Players in Development and Stress Responses. Annu. Rev. Cell Dev. Biol..

[16] Sunkar R., Li Y.-F., Jagadeeswaran G. (2012). Functions of microRNAs in plant stress responses. Trends Plant Sci..

[17] Sunkar R., Kapoor A., Zhu J.-K. (2006). Posttranscriptional induction of two Cu/Zn superoxide dismutase genes in Arabidopsis is mediated by downregulation of miR398 and important for oxidative stress tolerance. Plant Cell.

[18] Yamasaki H., Abdel-Ghany S.E., Cohu C.M., Kobayashi Y., Shikanai T., Pilon M. (2007). Regulation of copper homeostasis by micro-RNA in Arabidopsis. J. Biol. Chem..

[19] Abdel-Ghany S.E., Pilon M. (2008). MicroRNA-mediated systemic down-regulation of copper protein expression in response to low copper availability in Arabidopsis. J. Biol. Chem..

[20] Yang Y., Xu L., Hao C., Wan M., Tao Y., Zhuang Y., Su Y., Li L. (2024). The microRNA408-plantacyanin module balances plant growth and drought resistance by regulating reactive oxygen species homeostasis in guard cells. Plant Cell.

[21] Pilon M. (2017). The copper microRNAs. New Phytol..

[22] Wierzbicki A.T., Blevins T., Swiezewski S. (2021). Long Noncoding RNAs in Plants. Annu. Rev. Plant Biol..

[23] Chen L., Zhu Q.-H., Kaufmann K. (2020). Long non-coding RNAs in plants: Emerging modulators of gene activity in development and stress responses. Planta.

[24] Kęska K., Szcześniak M.W., Adamus A., Czernicka M. (2021). Waterlogging-stress-responsive LncRNAs, their regulatory relationships with miRNAs and target genes in cucumber (*Cucumis sativus* L.). Int. J. Mol. Sci..

[25] Pavlovic J., Kostic L., Bosnic P., Kirkby E.A., Nikolic M. (2021). Interactions of silicon with essential and beneficial elements in plants. Front. Plant Sci..

[26] Shao J.F., Che J., Yamaji N., Shen R.F., Ma J.F. (2017). Silicon reduces cadmium accumulation by suppressing expression of transporter genes involved in cadmium uptake and translocation in rice. J. Exp. Bot..

[27] Che J., Yamaji N., Shao J.F., Ma J.F., Shen R.F. (2016). Silicon decreases both uptake and root-to-shoot translocation of manganese in rice. J. Exp. Bot..

[28] Huang S., Ma J.F. (2020). Silicon suppresses zinc uptake through down-regulating zinc transporter gene in rice. Physiol. Plant..

[29] Bosnić D., Bosnić P., Nikolić D., Nikolić M., Samardžić J. (2019). Silicon and Iron differently Alleviate Copper Toxicity in *Cucumber* Leaves. Plants.

[30] Bosnić D., Nikolić D., Timotijević G., Pavlović J., Vaculík M., Samardžić J., Nikolić M. (2019). Silicon alleviates copper (Cu) toxicity in cucumber by increased Cu-binding capacity. Plant Soil.

[31] Li J., Leisner S.M., Frantz J. (2008). Alleviation of copper toxicity in *Arabidopsis thaliana* by silicon addition to hydroponic solutions. J. Am. Soc. Hortic. Sci..

[32] Khandekar S., Leisner S. (2011). Soluble silicon modulates expression of *Arabidopsis thaliana* genes involved in copper stress. J. Plant Physiol..

[33] Kim Y.-H., Khan A.L., Kim D.-H., Lee S.-Y., Kim K.-M., Waqas M., Jung H.-Y., Shin J.-H., Kim J.-G., Lee I.-J. (2014). Silicon mitigates heavy metal stress by regulating P-type heavy metal ATPases, *Oryza sativa* low silicon genes, and endogenous phytohormones. BMC Plant Biol..

[34] Flora C., Khandekar S., Boldt J., Leisner S. (2019). Silicon alleviates long-term copper toxicity and influences gene expression in *Nicotiana tabacum*. J. Plant Nutr..

[35] Zhu Y., Yin J., Liang Y., Liu J., Jia J., Huo H., Wu Z., Yang R., Gong H. (2019). Transcriptomic dynamics provide an insight into the mechanism for silicon-mediated alleviation of salt stress in cucumber plants. Ecotoxicol. Environ. Saf..

[36] Hernandez-Apaolaza L. (2022). Priming with Silicon: A Review of a Promising Tool to Improve Micronutrient Deficiency Symptoms. Front. Plant Sci..

[37] Coskun D., Deshmukh R., Sonah H., Menzies J.G., Reynolds O., Ma J.F., Kronzucker H.J., Bélanger R.R. (2019). The controversies of silicon’s role in plant biology. New Phytol..

[38] Yang W., Hu Y., Liu J., Rao X., Huang X., Guo X., Zhang J., Rensing C., Xing S., Zhang L. (2024). Physiology and transcriptomic analysis revealed the mechanism of silicon promoting cadmium accumulation in *Sedum alfredii* Hance. Chemosphere.

[39] Moy A., Czajka K., Michael P., Nkongolo K. (2024). Gene expression profiling of Jack Pine (*Pinus banksiana*) under copper stress: Identification of genes associated with copper resistance. PLoS ONE.

[40] Vaculík M., Lukačová Z., Bokor B., Martinka M., Tripathi D.K., Lux A. (2020). Alleviation mechanisms of metal(loid) stress in plants by silicon: A review. J. Exp. Bot..

[41] Liu Q., Zheng L., He F., Zhao F.-J., Shen Z., Zheng L. (2015). Transcriptional and physiological analyses identify a regulatory role for hydrogen peroxide in the lignin biosynthesis of copper-stressed rice roots. Plant Soil.

[42] Andrés-Colás N., Sancenón V., Rodríguez-Navarro S., Mayo S., Thiele D.J., Ecker J.R., Puig S., Peñarrubia L. (2006). The Arabidopsis heavy metal P-type ATPase HMA5 interacts with metallochaperones and functions in copper detoxification of roots. Plant J..

[43] Lee S., Jeon U.S., Lee S.J., Kim Y.-K., Persson D.P., Husted S., Schjørring J.K., Kakei Y., Masuda H., Nishizawa N.K. (2009). Iron fortification of rice seeds through activation of the nicotianamine synthase gene. Proc. Natl. Acad. Sci. USA.

[44] Guo W.-J., Meetam M., Goldsbrough P.B. (2008). Examining the specific contributions of individual Arabidopsis metallothioneins to copper distribution and metal tolerance. Plant Physiol..

[45] Huang S., Yamaji N., Ma J.F. (2024). Metal Transport Systems in Plants. Annu. Rev. Plant Biol..

[46] Li J., Wang W., Yuan J., Xu J., He L., Zhang X., Zhang H. (2021). Ubiquitin-independent proteasome system is required for degradation of Arabidopsis COPPER TRANSPORTER 2. Plant Sci..

[47] Andrés-Colás N., Perea-García A., de Andrés S.M., Garcia-Molina A., Dorcey E., Rodríguez-Navarro S., Pérez-Amador M.A., Puig S., Peñarrubia L. (2013). Comparison of global responses to mild deficiency and excess copper levels in Arabidopsis seedlings. Metallomics.

[48] Garcia-Molina A., Andrés-Colás N., Perea-García A., Neumann U., Dodani S.C., Huijser P., Peñarrubia L., Puig S. (2013). The Arabidopsis COPT6 transport protein functions in copper distribution under copper-deficient conditions. Plant Cell Physiol..

[49] Perea-García A., Andrés-Bordería A., Mayo de Andrés S., Sanz A., Davis A.M., Davis S.J., Huijser P., Peñarrubia L. (2016). Modulation of copper deficiency responses by diurnal and circadian rhythms in *Arabidopsis thaliana*. J. Exp. Bot..

[50] Sancenón V., Puig S., Mira H., Thiele D.J., Peñarrubia L. (2003). Identification of a copper transporter family in *Arabidopsis thaliana*. Plant Mol. Biol..

[51] Andrés-Colás N., Carrió-Seguí A., Abdel-Ghany S.E., Pilon M., Peñarrubia L. (2018). Expression of the Intracellular COPT3-Mediated Cu Transport Is Temporally Regulated by the TCP16 Transcription Factor. Front. Plant Sci..

[52] Yuan M., Li X., Xiao J., Wang S. (2011). Molecular and functional analyses of COPT/Ctr-type copper transporter-like gene family in rice. BMC Plant Biol..

[53] Bernal M., Casero D., Singh V., Wilson G.T., Grande A., Yang H., Dodani S.C., Pellegrini M., Huijser P., Connolly E.L. (2012). Transcriptome sequencing identifies SPL7-regulated copper acquisition genes FRO4/FRO5 and the copper dependence of iron homeostasis in Arabidopsis. Plant Cell.

[54] Robe K., Lefebvre-Legendre L., Cleard F., Barberon M. (2025). Four ZIPs contribute to Zn, Fe, Cu and Mn acquisition at the outer root domain. PLoS Genet..

[55] Yruela I. (2009). Copper in plants: Acquisition, transport and interactions. Funct. Plant Biol..

[56] Li Y., Iqbal M., Zhang Q., Spelt C., Bliek M., Hakvoort H.W.J., Quattrocchio F.M., Koes R., Schat H. (2017). Two *Silene vulgaris* copper transporters residing in different cellular compartments confer copper hypertolerance by distinct mechanisms when expressed in *Arabidopsis thaliana*. New Phytol..

[57] Shi K., Liu X., Zhu Y., Bai Y., Shan D., Zheng X., Wang L., Zhang H., Wang C., Yan T. (2020). MdWRKY11 improves copper tolerance by directly promoting the expression of the copper transporter gene MdHMA5. Hortic. Res..

[58] Deng F., Yamaji N., Xia J., Ma J.F. (2013). A member of the heavy metal P-type ATPase OsHMA5 is involved in xylem loading of copper in rice. Plant Physiol..

[59] Shabbir Z., Sardar A., Shabbir A., Abbas G., Shamshad S., Khalid S., Natasha, Murtaza G., Dumat C., Shahid M. (2020). Copper uptake, essentiality, toxicity, detoxification and risk assessment in soil-plant environment. Chemosphere.

[60] Rai S., Singh P.K., Mankotia S., Swain J., Satbhai S.B. (2021). Iron Homeostasis in Plants and its Crosstalk with Copper, Zinc, and Manganese. Plant Stress.

[61] Grotz N., Fox T., Connolly E., Park W., Guerinot M.L., Eide D. (1998). Identification of a family of zinc transporter genes from Arabidopsis that respond to zinc deficiency. Proc. Natl. Acad. Sci. USA.

[62] Ishimaru Y., Suzuki M., Kobayashi T., Takahashi M., Nakanishi H., Mori S., Nishizawa N.K. (2005). OsZIP4, a novel zinc-regulated zinc transporter in rice. J. Exp. Bot..

[63] Sheng H., Jiang Y., Rahmati M., Chia J.-C., Dokuchayeva T., Kavulych Y., Zavodna T.-O., Mendoza P.N., Huang R., Smieshka L.M. (2021). YSL3-mediated copper distribution is required for fertility, seed size and protein accumulation in Brachypodium. Plant Physiol..

[64] Chen C.-C., Chen Y.-Y., Tang I.-C., Liang H.-M., Lai C.-C., Chiou J.-M., Yeh K.-C. (2011). Arabidopsis SUMO E3 ligase SIZ1 is involved in excess copper tolerance. Plant Physiol..

[65] Yuan X., Liang R., Wang G., Ma S., Liu N., Gong Y., Mccouch S.R., Zhu H., Liu Z., Li Z. (2025). Design of rice with low cadmium accumulation in grain using single segment substitution line. New Crops.

[66] Chen H.-P., Wang P., Chang J.-D., Kopittke P.M., Zhao F.-J. (2021). Producing Cd-safe rice grains in moderately and seriously Cd-contaminated paddy soils. Chemosphere.

[67] Perea-García A., Andrés-Bordería A., Huijser P., Peñarrubia L. (2021). The Copper-microRNA Pathway Is Integrated with Developmental and Environmental Stress Responses in *Arabidopsis thaliana*. Int. J. Mol. Sci..

[68] Vaucheret H., Vazquez F., Crété P., Bartel D.P. (2004). The action of ARGONAUTE1 in the miRNA pathway and its regulation by the miRNA pathway are crucial for plant development. Genes Dev..

[69] Hu Y., Ji J., Cheng H., Luo R., Zhang J., Li W., Wang X., Zhang J., Yao Y. (2023). The miR408a-BBP-LAC3/CSD1 module regulates anthocyanin biosynthesis mediated by crosstalk between copper homeostasis and ROS homeostasis during light induction in Malus plants. J. Adv. Res..

[70] Zhang H., Zhao X., Li J., Cai H., Deng X.W., Li L. (2015). MicroRNA408 Is Critical for the HY5-SPL7 Gene Network That Mediates the Coordinated Response to Light and Copper. Plant Cell.

[71] Murata M., Begg G.S., Lambrou F., Leslie B., Simpson R.J., Freeman H.C., Morgan F.J. (1982). Amino acid sequence of a basic blue protein from cucumber seedlings. Proc. Natl. Acad. Sci. USA.

[72] Guss J.M., Merritt E.A., Phizackerley R.P., Freeman H.C. (1996). The Structure of a Phytocyanin, the Basic Blue Protein from Cucumber, Refined at 1.8 Å Resolution. J. Mol. Biol..

[73] Adrees M., Ali S., Rizwan M., Ibrahim M., Abbas F., Farid M., Zia-ur-Rehman M., Irshad M.K., Bharwana S.A. (2015). The effect of excess copper on growth and physiology of important food crops: A review. Environ. Sci. Pollut. Res..

[74] Beauclair L., Yu A., Bouché N. (2010). microRNA-directed cleavage and translational repression of the copper chaperone for superoxide dismutase mRNA in Arabidopsis. Plant J..

[75] Li J., Song Q., Zuo Z.-F., Liu L. (2022). MicroRNA398: A Master Regulator of Plant Development and Stress Responses. Int. J. Mol. Sci..

[76] Nikolić D., Bosnić D., Samardžić J. (2023). Silicon in action: Between iron scarcity and excess copper. Front. Plant Sci..

[77] Kim Y.-H., Khan A.L., Waqas M., Lee I.-J. (2017). Silicon Regulates Antioxidant Activities of Crop Plants under Abiotic-Induced Oxidative Stress: A Review. Front. Plant Sci..

[78] Trindade I., Capitão C., Dalmay T., Fevereiro M.P., dos Santos D.M. (2010). miR398 and miR408 are up-regulated in response to water deficit in *Medicago truncatula*. Planta.

[79] Fahad M., Tariq L., Muhammad S., Wu L. (2024). Underground Communication: Long Noncoding RNA Signaling in the Plant Rhizosphere. Plant Commun..

[80] Cho S.H., Coruh C., Axtell M.J. (2012). miR156 and miR390 regulate tasiRNA accumulation and developmental timing in *Physcomitrella patens*. Plant Cell.

[81] Mortazavi A., Williams B.A., McCue K., Schaeffer L., Wold B. (2008). Mapping and quantifying mammalian transcriptomes by RNA-Seq. Nat. Methods.

[82] Liao Y., Smyth G.K., Shi W. (2014). featureCounts: An efficient general purpose program for assigning sequence reads to genomic features. Bioinformatics.

[83] Robinson M.D., McCarthy D.J., Smyth G.K. (2010). edgeR: A Bioconductor package for differential expression analysis of digital gene expression data. Bioinformatics.

[84] Young M.D., Wakefield M.J., Smyth G.K., Oshlack A. (2010). Gene ontology analysis for RNA-seq: Accounting for selection bias. Genome Biol..

[85] Kanehisa M., Goto S. (2000). KEGG: Kyoto encyclopedia of genes and genomes. Nucleic Acids Res..

[86] Langmead B., Trapnell C., Pop M., Salzberg S.L. (2009). Ultrafast and memory-efficient alignment of short DNA sequences to the human genome. Genome Biol..

[87] Wen M., Shen Y., Shi S., Tang T. (2012). miREvo: An integrative microRNA evolutionary analysis platform for next-generation sequencing experiments. BMC Bioinform..

[88] Friedländer M.R., Mackowiak S.D., Li N., Chen W., Rajewsky N. (2012). miRDeep2 accurately identifies known and hundreds of novel microRNA genes in seven animal clades. Nucleic Acids Res..

[89] Wu H.-J., Ma Y.-K., Chen T., Wang M., Wang X.-J. (2012). PsRobot: A web-based plant small RNA meta-analysis toolbox. Nucleic Acids Res..

[90] Zhou L., Chen J., Li Z., Li X., Hu X., Huang Y., Zhao X., Liang C., Wang Y., Sun L. (2010). Integrated Profiling of MicroRNAs and mRNAs: MicroRNAs Located on Xq27.3 Associate with Clear Cell Renal Cell Carcinoma. PLoS ONE.

[91] Varkonyi-Gasic E., Wu R., Wood M., Walton E.F., Hellens R.P. (2007). Protocol: A highly sensitive RT-PCR method for detection and quantification of microRNAs. Plant Methods.

[92] Remans T., Smeets K., Opdenakker K., Mathijsen D., Vangronsveld J., Cuypers A. (2008). Normalisation of real-time RT-PCR gene expression measurements in *Arabidopsis thaliana* exposed to increased metal concentrations. Planta.

[93] Timotijević G., Milisavljević M., Nikolić D., Milovanović B., Nikolić D., Nikolic M., Samardžić J. (2015). Establishment and In-house validation of stem-loop RT-PCR method for microRNA398 expression analysis. Genetika.

