# Supplementary material for: Silicon Modulates the Expression of Copper-Responsive Transporters and Non-Coding RNAs in Cucumber Subjected to Copper Stress
Source: Plants (Basel). 2026 Oct 18;15(20):3189. doi: 10.3390/plants15203189 (PMC13708774; doi:10.3390/plants15203189)
Supplement: Supplementary file 1 [file plants-15-03189-s001.zip › Table S7.pdf]

miRNA ID	Precursor sequence (5’→3’)	Mature seguence (5’→3’)	MFE ($DeltaG_{mfe}$, kcal/mol)	Ensemble FE ($DeltaG_{ensemble}$, kcal/mol)	miRNA Arm
**novel_1**	uggucguccugaaaacacauguaauuacguuguuuacguagcauucaacauguguucucaggucgccccug	uggucguccugaaaacacaug	-27.40	-28.77	5p
**novel_11**	uggagaagcagggcacgugcauuacucacucguggaaguccuaacacaacaccaccgguggcugugucugagugaggguuguucaugugccccucuucuccauc	caugugccccucuucuccauc	-51.20	-53.12	3p
**novel_12**	uuccacagcuuucuugaacuuucuuugcucuucccccucuucucaugcucauuuguauguaaaaucucucuuagauuuuacccaaaauuuuaaauuuaaaaaaaaagacuuuggaaguucaagaaagcugugggaga	guucaagaaagcugugggaga	-45.50	-47.34	3p
**novel_15**	uggagaagcagggcacgugcauggcacaugccauauauacacaaacacaugauacacacacccuccacugcacgugcucccuuucuccaac	cacgugcucccuuucuccaac	-32.40	-34.11	3p
**novel_18**	aagcucaggagggauagcgccgucaucguuucgauucaccucccucgacaucaucaucuuauuauuucucggcgcuauccauccugaguuucc	cgcuauccauccugaguuucc	-36.10	-37.58	3p
**novel_21**	ugaagcugccagcaugaucugaggaacgcuaaaacauuugguuaaagauugguuucuuccaucaggucaucuugcagcuuca	ugaagcugccagcaugaucugag	-34.10	-35.48	5p
**novel_25**	gaacuuauuagauaccacagaagcaaugguaucuaauaaguuuu	caaugguaucuaauaaguuuu	-19.80	-20.15	3p
**novel_28**	ucgcaggagagauggcaccaacuauuuuaacaacacaauaucuaauuaaaguaguuggugucaucucacugcgac	ucgcaggagagauggcaccaac	-41.10	-41.43	5p
**novel_29**	gauuucgcuccaaaaugucucauauacaaugaagaucauuggaaaugcaaugaaaguucuacauugguuuuagauaagaagaugaucguaaguaagugagaacaacuauaucucuauugauacgagaucuuuuggaguaaaacuaa	cgagaucuuuuggaguaaaacuaa	-37.20	-39.17	3p

**novel_3**	ggaauguugucuggugcgagaucuuucauuucuuuugaauuuuugaaacccuuaauucaaauuacaacucuuuacuuuugaaugauuucggaccaggcuucauuccc	uucggaccaggcuucauuccc	-37.90	-38.87	3p
**novel_33**	aagcucaggagggauagcgccaauuaauaguacaaaauaauuuucguucaaagcuucacacuuugcuuuuuguuaauggcgcuauccauccugaguuuc	cgcuauccauccugaguuuc	-41.30	-42.72	3p
**novel_38**	ucaagcugccagcaugaucuaaucuuaaucucuccuuauuuuacuuuugaauuuuguaaauauugagguuaaagaucagaucauauggcaguuuuaccu	ucaagcugccagcaugaucuaau	-33.00	-35.00	5p
**novel_46**	ucagauucuaucgauaauaaagaucgauaaacauugauagacacuaauauuugaucuaucaauauaucuaucaaugcacuuuguugauagaauaugaaa	cuuuguugauagaauaugaaa	-27.00	-28.05	3p
**novel_47**	cgagugaauggacgauacgaauaauuacuguacauaaaacuaaaauuaacuuuagauaguauagcauucuauuuacuugugugaacugauuaaucaauauuuauaauuaggcauccaaauaaacgaauaaaaucaguucacacaaguaaauagaaugcuauacuaucuaaaguuaauuuuaguuuuauguacgguaauuauucagaucguccuuucacucgga	auucagaucguccuuucacucgga	-133.20	-134.86	3p
**novel_48**	cgucguggaaaguuuacuaauuaauuuguuauuuuaccuuuccgcgacguuugaggacaucacgucguggaaagucgaaacguuuuauuucauaggagacuuuuugcgaugucuaucucccaaacgucacgaaaaugugaauauuuaauuuguuauuucgacuuuccaugacgugauguccucaaacguugcggaaaggugaacauuaaauuuaauauuuggacuuuuugcgacgug	aauauuuggacuuuuugcgacgug	-117.20	-119.68	3p
**novel_49**	ugugaacuguauaauuaauauuacgguuuauuuaaauaucauucaauuuuaucuucaaacgaaauuaaauaaacguuuauuuauuaugcuagauucacgcc	ugugaacuguauaauuaauauuac	-18.30	-20.80	5p
**novel_51**	aauggugggggaacucuugacguguauuugguguggcaucaggaguuuccccaccauuuc	aaugguggggaacucuugacgugu	-31.20	-32.48	5p

**novel_52**	uuuccuaaaccagucuuuucuaaaaacuuacaucguuuuccuuaaaaaaauuucuacgauuucuuguuaucaaaucucggacuuccaaagauauugaucacucaccucaagaaucuagacauaacagacuuccaaaaaguucuagauuccaucguaaauuuuuuuuuauggaaaacgguguaaguuuuuagaaaagacugguuuaggaaauc	uagaaaagacugguuuaggaaauc	-90.30	-91.80	3p
**novel_58**	augagccaagaaugacuugccggccgacuuucgacaccugucguucucugguucggcgcggucuucuucggcucuugu	cggcgcggucuucuucggcucuugu	-38.20	-38.73	3p
**novel_59**	ucauacuguaauucuuaaauuggcgucguuuuuaaaaagauggaucuaaauacacaaaaauaaaaguauauguauuaugacauuguuaauuguuuuaauguuuuauuuauaaauauaaucgauuaauucuuuuaauguuuuaauuaaauuuaaauuuuauauauuuggauccaucuuuuuuaaaauggugucgauuuaagacuugcaguaugaau	ucauacuguaauucuuaaauuggc	-69.40	-72.85	5p
**novel_61**	cuaggccaucucauucuaaguuucucuugaaagggaaugaucaguucuugcaguaguuaggucggcaaguagucagaagccccagauugaccuaguaggcugaguuggaaaggaagggagaacucacuucugguuggguucguaagaaggcuggauuuccgcgaugauucgugugcagugauuauuucuuucuucuuauugguuauugggaguugauaggucuguga	uauugggaguugauaggucuguga	-59.00	-63.99	3p
**novel_62**	ccaaaaaugacuugccuacuaugcaaacuauguguuguagagcuuguauuagacguuuguauaucaggcaaucauuuuuggcc	ccaaaaaugacuugccuacu	-29.10	-29.90	5p
**novel_63**	gccaaacucucggggaaaaaccucauguuccccaguggagucgcca	uccccaguggagucgcca	-14.00	-14.36	3p
**novel_65**	guauugccuugagaugucaugauaucuuuagauguucuucaaaugagaaagagaugaaaguuagagauuaucccacugggcgugcca	aucccacugggcgugcca	-15.30	-17.86	3p
**novel_9**	gggcggaucuuuuuuggcagguggauucaucuaaauuaaaauugaucugccaaaagagacuugcccug	ugccaaaagagacuugcccug	-33.70	-34.03	3p
